# Efficacy of Lifting Threads Composed of Poly(L‐Lactide‐Co‐ε‐Caprolactone) Copolymers Coated With Hyaluronic Acid: A Long‐Term Study on Biorevitalizing Properties in Skin Remodeling

**DOI:** 10.1111/jocd.70077

**Published:** 2025-03-12

**Authors:** Pavel Burko, George Sulamanidze, Dmitriy Nikishin

**Affiliations:** ^1^ Section of Human Anatomy, Department of Biomedicine, Neurosciences and Advanced Diagnostics (BiND) University of Palermo Palermo Italy; ^2^ Headquarter of the APTOS LLC Tbilisi Georgia; ^3^ Russian Office of the APTOS LLC Moscow Russia

**Keywords:** biorevitalization, hyaluronic acid, lifting threads, non‐surgical facelift, skin remodeling

## Abstract

**Background:**

Facial thread lifting, which is popular in aesthetic medicine because of its minimal invasiveness, has led to advancements in the use of biodegradable polymers such as poly(L‐lactide‐co‐ε‐caprolactone) (P(LA/CL)) and its hyaluronic acid‐coated variant (P(LA/CL)‐HA). These developments enhance biocompatibility and efficacy, offering prolonged benefits through better biostimulation and tissue integration.

**Methods:**

A controlled experiment involving five 4‐month‐old female pigs compared the effectiveness of P(LA/CL) and P(LA/CL)‐HA threads over six months. After being implanted into the subcutaneous layer parallel to the skin of each pig, the threads were evaluated through histological analysis at intervals (7, 21, 30, 90, and 180 days) to assess changes in dermal and hypodermal structures, collagen types, elastin levels, and tissue integration via stains such as hematoxylin and eosin (H&E), Weigert‐Van Gieson, and Sirius Red.

**Results:**

P(LA/CL) and P(LA/CL)‐HA threads significantly led to dermal and hypodermal remodeling. By day 180, a comparative analysis via the Wilcoxon test revealed that P(LA/CL)‐HA significantly outperformed P(LA/CL) in most histological indicators because of the biostimulatory effects of hyaluronic acid.

**Conclusions:**

Our study investigated the effects of two types of bioabsorbable threads, P(LA/CL) and P(LA/CL)‐HA, on skin remodeling over 180 days in a porcine model. These results highlight the significant role of thread composition in tissue remodeling and suggest that incorporating HA could optimize therapeutic and aesthetic outcomes. Further clinical research is necessary to confirm these findings in human subjects.

## 1 Introduction

Facial thread lifting represents a minimally invasive procedure noted for its excellent aesthetic outcomes. This treatment is generally associated with minimal serious complications, underscoring its safety and effectiveness [1]. Historically, nonabsorbable cogged threads have been supplanted by absorbable threads, which are deemed safer, facilitate modifications, and are associated with a significantly reduced risk of paresthesia and thread extrusion [2, 3].

Currently, absorbable threads are crafted from biodegradable synthetic polymers, including polydioxanone (PDO) or poly‐p‐dioxanone (PPDO), poly‐L‐lactic acid (PLLA), polyglycolic acid (PGA), polylactic‐co‐glycolic acid (PLGA), polylactic acid‐caprolactone (PLACL), and poly(ε‐caprolactone) (PCL) [4, 5, 6, 7]. In recent years, the field has also included the introduction of poly(L‐lactide‐co‐ε‐caprolactone) (P(LA/CL)), a copolymer of L‐lactic acid (LA) and ε‐caprolactone (CL). This material, which is applied in various domains of tissue engineering and regenerative medicine, as well as aesthetic medicine, exhibits excellent biocompatibility, safety, and tolerability and is suitable for applications requiring prolonged duration due to its slower degradation rate [8, 9, 10]. The degradation timeline for these threads typically spans 9–12 months, providing adequate support for tissue remodeling and potentially extending the lifting effect [11]. Clinical studies have shown that the effects of P(LA/CL) threads can persist beyond 18 months because of their collagen‐stimulating properties, which are superior to those of previous generations [10].

As aesthetic medicine has rapidly advanced, addressing a significant gap in prognostic effectiveness is critical. When threads are selected for facial thread lifting, it is imperative to consider not only the threads’ ability to provide an immediate lifting effect but also their potential for promoting structural skin remodeling. The lack of biorevitalizing properties of these threads may limit the long‐term efficacy of lifting procedures, potentially resulting in failure to meet the expectations of aesthetic medicine practitioners and their patients. To improve therapeutic outcomes, thread lifting is frequently combined with hyaluronic acid injections, which have been shown to improve clinical results [12, 13]. With this synergistic approach, APTOS LLC (Tbilisi, Georgia) has developed hyaluronic acid‐coated threads that aim to streamline the lifting process and minimize post‐procedure adverse effects, thus enhancing overall patient outcomes [14].

The positive impact of P(LA/CL) threads on the production of collagen, which is crucial for skin rejuvenation, is well documented [10]. However, it is essential to recognize that an effective evaluation of structural skin remodeling should extend beyond simply assessing the impacts on collagenogenesis. A thorough evaluation must also consider the thread's selective effects on stimulating type I and III collagen, enhancing elastogenesis, altering the cellular composition, and influencing the morphometric characteristics of vessels. Additionally, a comprehensive morphological evaluation may require the consideration of further parameters to fully appreciate the intervention's scope and efficacy.

In the experiments, lifting threads developed by APTOS LLC (Tbilisi, Georgia), which are composed of P(LA/CL) and coated with hyaluronic acid, were employed. The primary objective of this study was to evaluate and compare the efficacy of P(LA/CL)‐HA threads and P(LA/CL) threads over a six‐month period, with a specific focus on their impact on skin remodeling.

## 2 Materials and Methods

### 2.1 Experimental Animals

The experimental study was conducted on five 4‐month‐old female pigs, each weighing 40 kg at the onset of the experiment, of the Large White breed. The pigs were maintained under optimal conditions at a temperature of 21°C ± 2°C, a relative humidity of 30%–60%, and a 12 h light/12 h dark cycle starting at 7 a.m., and were fed food and water ad libitum.

### 2.2 Experimental Process

After one week of acclimatization to the environment, the pigs were numbered randomly. The procedure of thread placement was executed under both inhalational and intravenous anesthesia within the confines of a sterile surgical environment. Systemic analgesics, which are commonly administered in various clinical and veterinary settings, were employed throughout the investigation to ensure optimal pain management. The surgical area was shaved and disinfected with 70% alcohol. Five threads of each type were implanted into the torso and limbs (forelimbs and hindlimbs) of each pig via an 18‐G straight needle inserted into the subcutaneous layer parallel to the skin. Right‐sided access: 5 puncture points. Implantation of absorbable threads P(LA/CL)‐HA APTOS LLC (Tbilisi, Georgia), 5 units. Left‐sided access: 5 puncture points. Implantation of P(LA/CL) threads APTOS LLC (Tbilisi, Georgia), 5 units. Aseptic adhesive seals were applied to all the access points. Each suture used in the procedure measured 15 cm in length and conformed to the USP 2‐0 standard for non‐barbed threads. The surgical procedure was completed without complications. Following the implantation of lifting threads, skin samples incorporating subcutaneous tissue were excised and subjected to comprehensive histological analysis at five predetermined intervals: 7, 21, 30, 90, and 180 days post‐procedure. From each 15 cm long sample, three fragments were extracted for evaluation—one from each extremity and one from the central section—to facilitate a detailed assessment of tissue responses over time. To establish a baseline for comparison, adjacent skin that had not undergone any treatment was designated as the control. Each pig underwent the same procedure at 1 week, 21 days, 1 month, 3 months, and 6 months after the threading procedure.

### 2.3 Histologic Analysis

The tissue samples were fixed in 7% neutral buffered formalin for 24 h, which halted the biochemical reactions within the tissues. Following fixation, the biopsy samples were dehydrated via an ascending series of alcohols and subsequently infiltrated with organic solvents before being embedded in hot liquid paraffin. As the paraffin cooled, it solidified, thus providing structural support to the tissue. The prepared tissues were sectioned at full thickness into slices measuring 5–7 μm in thickness via a microtome. A minimum of three sections from each fragment were carefully mounted onto glass slides for subsequent microscopic analysis. These sections were subjected to a series of processes, including dewaxing, followed by staining. The following staining protocols were used to enhance histological differentiation: (1) hematoxylin and eosin (H&E) staining, (2) Weigert‐Van Gieson staining, and (3) Sirius Red staining. A digital microscope, outfitted with a Sony sensor and capable of delivering a resolution of 12 megapixels, was employed to scrutinize these stained sections via both standard light microscopy and polarized light microscopy techniques. Throughout the analysis, the image acquisition strategy involved capturing a series of detailed photographs: five images at a magnification of 40× for each glass slide, 10 images at a magnification of 100× per glass slide (equally divided between the dermis and hypodermis), and an additional 10 images at a magnification of 200× per glass slide (again, divided equally between the dermis and hypodermis). Morphological evaluations were conducted on the basis of the cellular composition, dermal thickness, thickness of the fibrous sheath, and morphometric characteristics of the vessels, including the diameter of the blood vessels and the relative vascular bed area. Additionally, assessments were made of the fibrocyte count, quantity of type I and III collagen in the dermis and hypodermis, ratio of type I/III collagen, collagen density, and elastin levels in the dermis and hypodermis. These evaluations were performed via the software programs ImageView v.3.7.7 and HistMorph v.2.3.

### 2.4 Statistical Evaluation

Basic statistical calculations were performed via the software package Statistica v.7. In the first stage of the statistical analysis, descriptive statistics were calculated, including mean values with the standard error (M ± m), medians, and quartiles (25% and 75%), as well as the minimum and maximum values for each day studied and for all days combined (Tables S1, S2, S3). The subsequent stage of the statistical investigation involved an analysis of the day‐to‐day dynamics of each parameter by type of thread individually. The data comparison was conducted via non‐parametric statistics, employing the Wilcoxon test for related samples. The results were considered statistically significant at *p* < 0.05, and a tendency toward a significant difference was suggested for 0.05 < *p* < 0.1, indicating that significance might be established with an increased number of samples analyzed. In conclusion, a comparative analysis was performed via the Wilcoxon test to compare both threads against each other and against the control for each studied day and across all days combined.

## 3 Results

### 3.1 Tissue Changes Due to P(LA/CL) Threads

**FIGURE 1:**
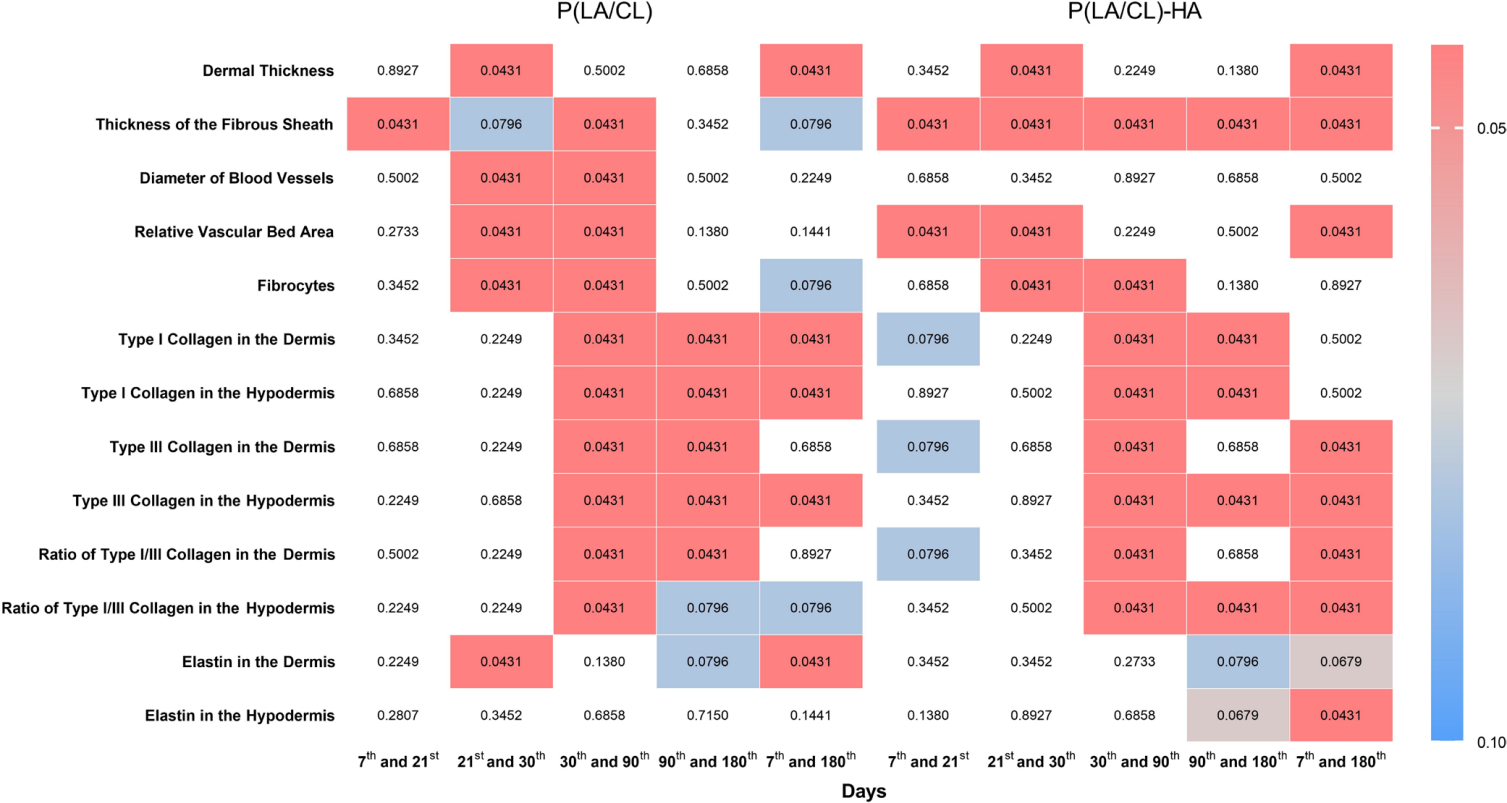
The heatmap represents the data across different indicators and time intervals for the P(LA/CL) and P(LA/CL)‐HA threads. Each cell's color intensity varies according to the *p*‐value, providing a visual representation of significance levels at various points. The results were considered statistically significant at *p* < 0.05, and a tendency toward a significant difference was suggested for 0.05 < *p* < 0.1, indicating that significance might be established with an increased number of samples analyzed. The non‐colored cells indicate a *p*‐value outside the defined range.

In this study, we employed nonparametric statistics via the Wilcoxon test for related samples to analyze the day‐to‐day dynamics of various biological indicators after the implantation of P(LA/CL) threads (Figure 1).

Our findings indicate that the most pronounced changes occurred between days 30 and 90. During this period, nearly all analyzed indicators exhibited statistically significant differences, except for dermal thickness and elastin density in both the dermis and hypodermis layers. This finding suggests that this interval may be particularly critical for observing the peak effects of P(LA/CL) on tissue structure and function, potentially capturing the most active phase of the biological response or therapeutic intervention.

Moreover, long‐term comparative analysis from day 7 to day 180 revealed significant changes in dermal thickness, type I collagen density in both the dermis and hypodermis layers, type III collagen levels in the hypodermis, and elastin density in the dermis. These changes are indicative of substantial tissue remodeling over the duration of the study. Additionally, trends toward statistically significant differences were observed in the thickness of the fibrous sheath, fibrocyte count, and ratio of type I to type III collagen in the hypodermis, suggesting that these indicators approach statistical significance and might show definitive differences with increasing sample sizes or over extended observation periods.

Conversely, other indicators did not demonstrate statistically significant changes over the same extended period (days 7–180), highlighting the variable nature of tissue responses to P(LA/CL). This variability underscores the complexity of biological adaptations and responses, which may not progress uniformly across all tissue components or follow the same temporal patterns.

### 3.2 Tissue Changes Due to P(LA/CL)‐HA Threads

In the evaluation of P(LA/CL)‐HA thread impact over time, our statistical analysis also employed the Wilcoxon test to discern changes between various time intervals (Figure 1). These thresholds provided a robust framework for evaluating the dynamism and efficacy of the tissue responses engendered by the threads.

The most notable changes occurred within the timeframe from days 30 to 90, when almost all analyzed indicators exhibited statistically significant differences, except for dermal thickness, blood vessel diameter, relative vascular bed area, and elastin levels in both the dermis and hypodermis layers. This period is highlighted as the peak phase for biological responses, where substantial tissue alterations are precipitated by the threads.

Over the extended duration from the first post‐implantation time point on day 7 to day 180, significant differences were observed in several parameters. These include the dermal thickness, thickness of the fibrous sheath, relative vascular bed area, type I collagen density, and ratios of type I/III collagen in both the dermis and hypodermis layers. Additionally, elastin density in the hypodermis was significantly increased, whereas elastin levels in the dermis tended to differ significantly. The other parameters did not significantly change over these intervals.

### 3.3 Comparative Analysis of P(LA/CL) and P(LA/CL)‐HA Threads on Various Histological Indicators

**FIGURE 2:**
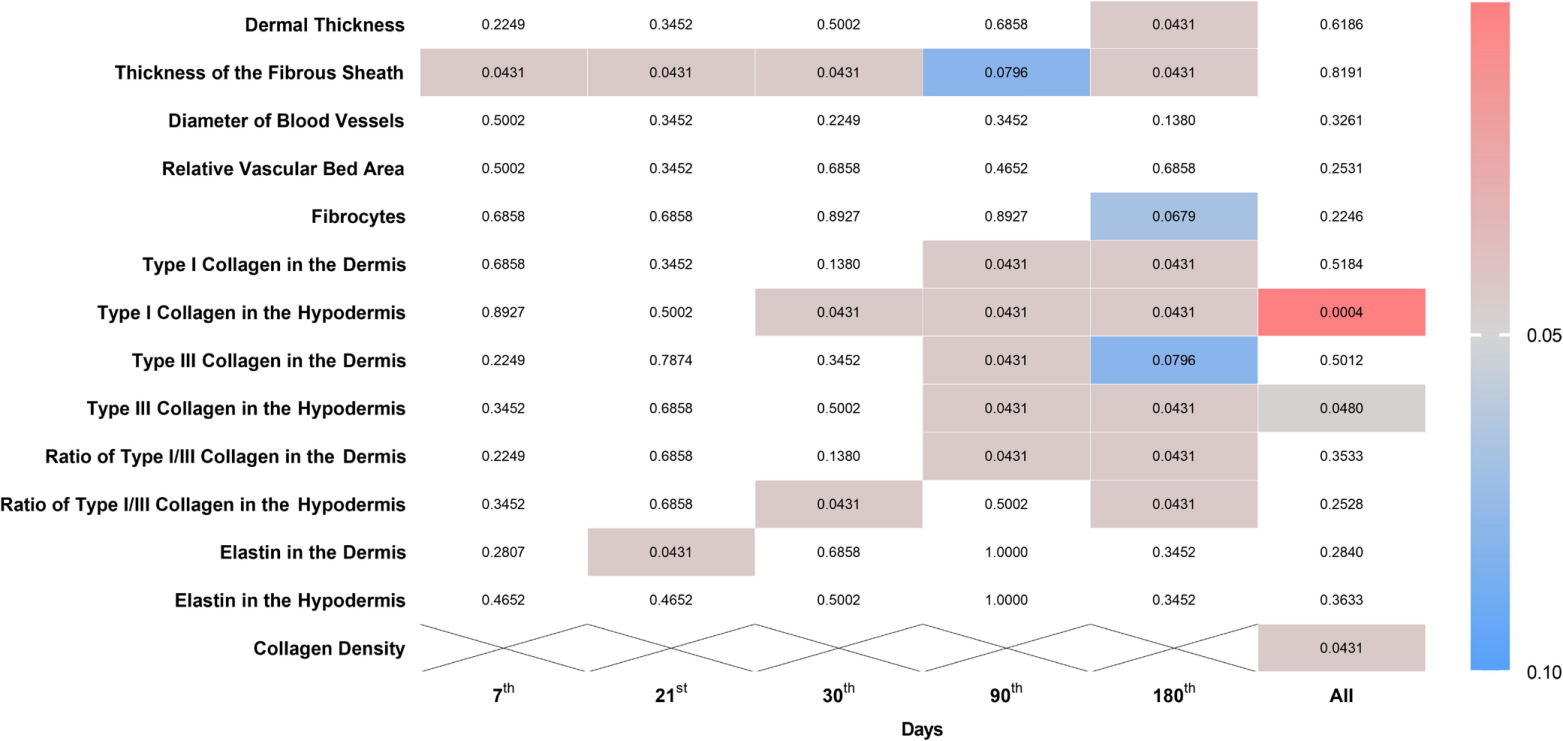
The heatmap represents the data across different indicators and days comparing P(LA/CL) and P(LA/CL)‐HA threads. Each cell's color intensity varies according to the *p*‐value, providing a visual representation of significance levels at various points. The results were considered statistically significant at *p* < 0.05, and a tendency toward a significant difference was suggested for 0.05 < *p* < 0.1, indicating that significance might be established with an increased number of samples analyzed. The non‐colored cells indicate a *p*‐value outside the defined range.

**FIGURE 3:**
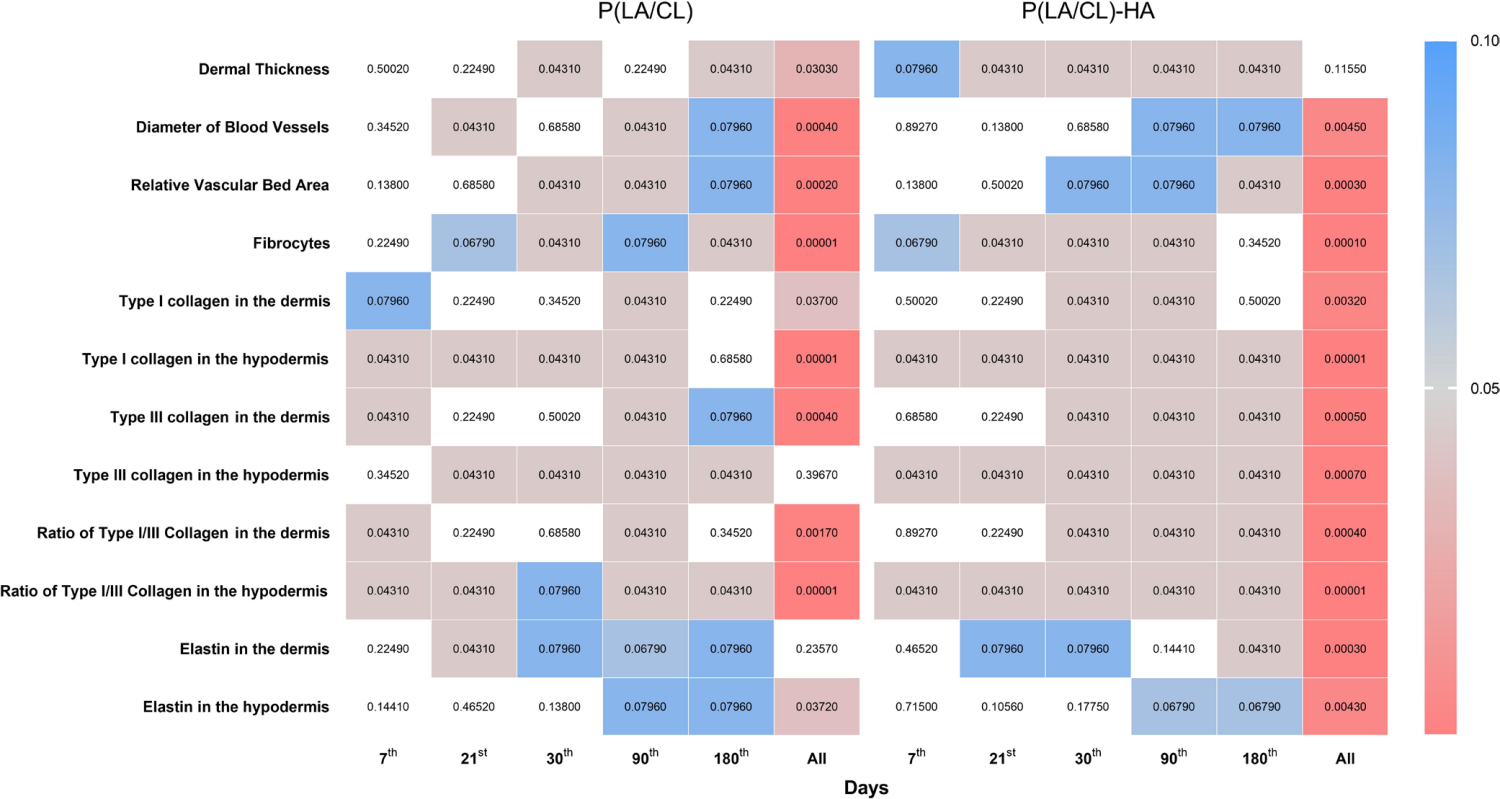
The heatmap represents the data across different indicators and days, comparing P(LA/CL) and P(LA/CL)‐HA threads with the control for each of them separately. Each cell's color intensity varies according to the *p*‐value, providing a visual representation of significance levels at various points. The results were considered statistically significant at *p* < 0.05, and a tendency toward a significant difference was suggested for 0.05 < *p* < 0.1, indicating that significance might be established with an increased number of samples analyzed. The non‐colored cells indicate a *p*‐value outside the defined range.

A comparative analysis utilizing the Wilcoxon test was performed to evaluate two types of threads, specifically P(LA/CL) and P(LA/CL)‐HA. This analysis was conducted both within the groups of threads and against a control group for each day of the study, as well as aggregately across the entire study period (Figures 2, 3).

According to the obtained data, when P(LA/CL)‐HA threads were used on day 180, a statistically significant difference was observed in most indicators, such as dermal thickness, thickness of the fibrous sheath, type I collagen levels in both the dermis and hypodermis layers, type III collagen density in the hypodermis, and the ratio of type I to type III collagen in the dermis, compared with similar parameters when P(LA/CL) threads were used. For these specified parameters, the values for P(LA/CL)‐HA were significantly greater than those for P(LA/CL). A statistically significant difference was also observed in the ratio of type I/III collagen in the hypodermis between P(LA/CL) and P(LA/CL)‐HA; however, unlike the parameters listed previously, this indicator was significantly greater for P(LA/CL) than for P(LA/CL)‐HA. Additionally, there was a trend toward a significant difference in fibrocyte count and type III collagen density in the dermis, with values on day 180 being greater for P(LA/CL) than for P(LA/CL)‐HA.

#### 3.3.1 Dermal Thickness

*Control* vs. *P(LA/CL)*. The dermal thickness when P(LA/CL) threads were used was lower than the control values on days 7 and 21. However, an increase in this indicator was observed by day 30, and on days 30, 90, and 180, the values exceeded those of the control, with the differences being statistically significant on days 21 and 180.

*Control* vs. *P(LA/CL)‐HA*. The dermal thickness when P(LA/CL)‐HA threads were used was lower than the control values on days 7 and 21, but an increase in this indicator was observed by day 30, and the values exceeded the control values on days 30, 90, and 180. The difference was statistically significant on days 21, 30, 90, and 180. On day 7, there was a trend toward statistically significant differences.

*P(LA/CL)* vs. *P(LA/CL)‐HA*. The dermal thickness increased throughout the study days when both P(LA/CL)‐HA and P(LA/CL) were used. Notably, on days 7 and 21, this parameter was lower with P(LA/CL)‐HA threads than with P(LA/CL), but by day 30, the measurements equalized for both thread types, and by day 180, the dermal thickness in P(LA/CL)‐HA was significantly greater than that in P(LA/CL).

**FIGURE 4:**
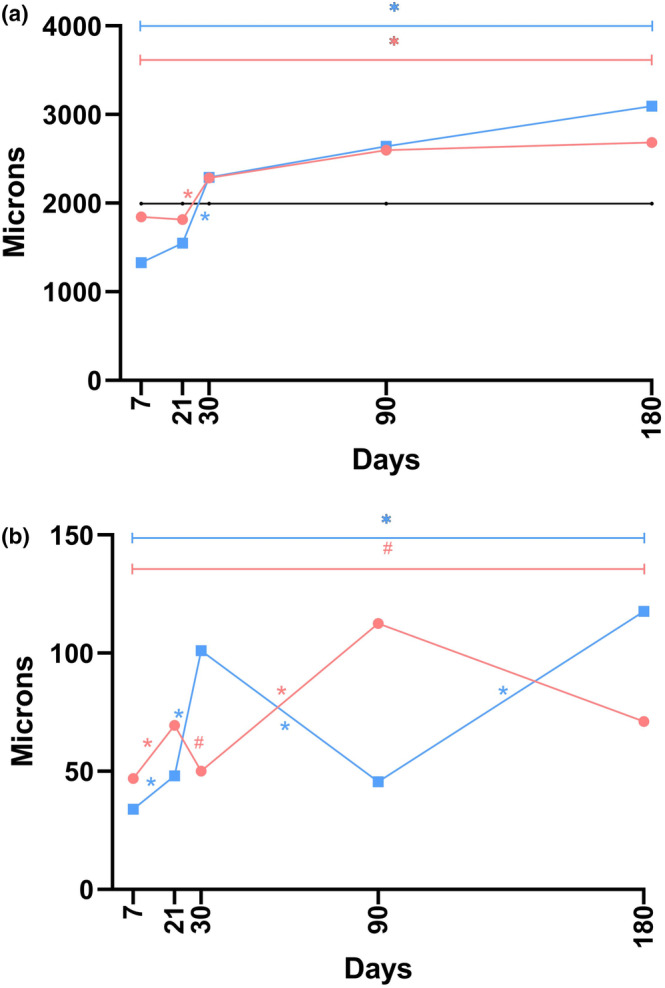
The linear graphs display data across the dermal thickness (a) and the thickness of the fibrous sheath (b) at five time points during the post‐implantation examination, constructed from median values. In the graphs, *black* represents the control, *blue* indicates P(LA/CL)‐HA, and *red* specifies P(LA/CL). The results were considered statistically significant at *p* < 0.05 (* for 0.01 ≤ *p* < 0.05), and a tendency toward a significant difference was suggested for 0.05 < *p* < 0.1 (#), indicating that significance might be established with an increased number of samples analyzed.

The data from the histological analysis of dermal thickness are visually displayed in Figures 4 and S1.

#### 3.3.2 Thickness of the Fibrous Sheath

*P(LA/CL)* vs. *P(LA/CL)‐HA*. The thickness of the fibrous sheath significantly differed throughout the study period between P(LA/CL) and P(LA/CL)‐HA. Notably, on days 7, 21, and 90, this indicator for P(LA/CL) exceeded the values observed with P(LA/CL)‐HA. Conversely, on days 30 and 180, the thickness was significantly greater when P(LA/CL)‐HA was used than when P(LA/CL) was used.

The histological analysis data pertaining to the thickness of the fibrous sheath are graphically illustrated in Figures 4 and S2.

#### 3.3.3 Diameter of Blood Vessels

*Control* vs. *P(LA/CL)*. The diameters of blood vessels when P(LA/CL) was used were greater than the control values on all days except for day 30, during which a decrease in this indicator was observed with the use of P(LA/CL). Statistically significant differences from the control were noted on day 21, and a trend toward a statistically significant difference was observed on day 180.

*Control* vs. *P(LA/CL)‐HA*. The diameters of the blood vessels in response to P(LA/CL)‐HA were greater than the control values on all days; however, on day 7, the median value of this parameter was slightly (but not significantly) lower than that of the control. No statistically significant differences were observed for this parameter, although a trend toward statistically significant differences was noted on days 90 and 180.

*P(LA/CL)* vs. *P(LA/CL)‐HA*. No statistically significant differences were noted in the diameters of the blood vessels. Notably, when P(LA/CL) threads were used, this parameter exceeded the corresponding values for P(LA/CL)‐HA on days 7, 21, and 180. However, on days 30 and 90, the values were greater for P(LA/CL)‐HA.

**FIGURE 5:**
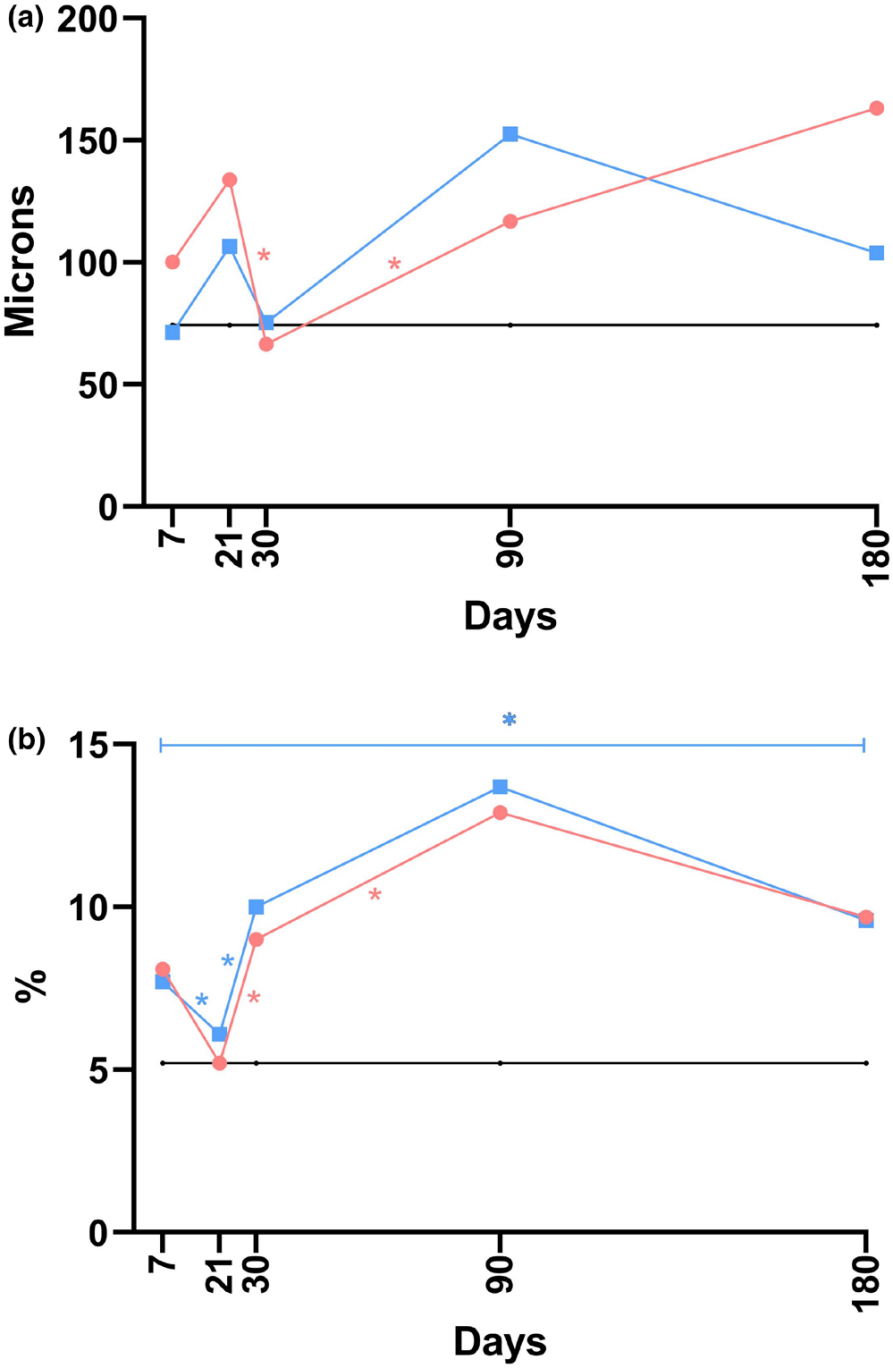
The linear graphs visually represent data pertaining to the diameter of blood vessels (a) and the relative vascular bed area (b) at five distinct time points during the post‐implantation period, utilizing median values for construction. Within these graphs, the *black* line denotes the control group, *blue* signifies the P(LA/CL)‐HA threads, and *red* is indicative of the P(LA/CL) threads. The results that reached a *p*‐value of less than 0.05 were deemed statistically significant (* denoting 0.01 ≤ *p* < 0.05).

Data from the histological examination, specifically concerning the diameter of the blood vessels, are graphically depicted in Figures 5 and S3.

#### 3.3.4 Relative Vascular Bed Area

*Control* vs. *P(LA/CL)*. The relative vascular bed area values, when P(LA/CL) was used, exceeded the control values on days 7, 30, 90, and 180, with statistically significant differences noted on days 30 and 90, and a trend toward a statistically significant difference was observed on day 180. On day 21, the values of this parameter when P(LA/CL) was used were nearly identical to those of the control.

*Control* vs. *P(LA/CL)‐HA*. The values of the relative vascular bed area when P(LA/CL)‐HA was used exceeded the control values throughout the entire analysis period. On day 180, this difference was statistically significant, and on days 30 and 90, there was a trend toward statistically significant differences.

*P(LA/CL)* vs. *P(LA/CL)‐HA*. For the relative vascular bed area, no statistically significant differences were observed between P(LA/CL) and P(LA/CL)‐HA. Notably, on day 7, this indicator was slightly greater for P(LA/CL). However, on days 21, 30, and 90, it was greater for P(LA/CL)‐HA than for P(LA/CL). By day 180, the values were nearly equal for both types of threads, with a marginal predominance observed for P(LA/CL).

The results of the histological examination, which focused on the relative vascular bed area, are graphically presented in Figures 5 and S4.

#### 3.3.5 Fibrocytes

*Control* vs. *P(LA/CL)*. Throughout the entire study period, the fibrocyte count was greater when P(LA/CL) was used than when the control was used. This difference was statistically significant on days 30 and 180, and there was a trend toward a statistically significant difference on days 21 and 90.

*Control* vs. *P(LA/CL)‐HA*. During the entire duration of the study, the fibrocyte count recorded from samples treated with P(LA/CL)‐HA consistently exceeded that of the control group. This disparity reached statistical significance on days 21, 30, and 90. Additionally, initial observations suggested a trend toward statistical significance as early as day 7.

*P(LA/CL)* vs. *P(LA/CL)‐HA*. For the fibrocyte count, a trend toward a statistically significant difference between P(LA/CL) and P(LA/CL)‐HA was observed on day 180. Notably, on day 30, both types of threads exhibited a significant increase compared with those on day 21, followed by a sharp decrease by day 90 compared with those on day 30. On every analyzed day, except for day 30, this indicator was slightly greater for P(LA/CL) than for P(LA/CL)‐HA. Conversely, on day 30, a noticeable increase was observed with P(LA/CL)‐HA compared with P(LA/CL).

**FIGURE 6:**
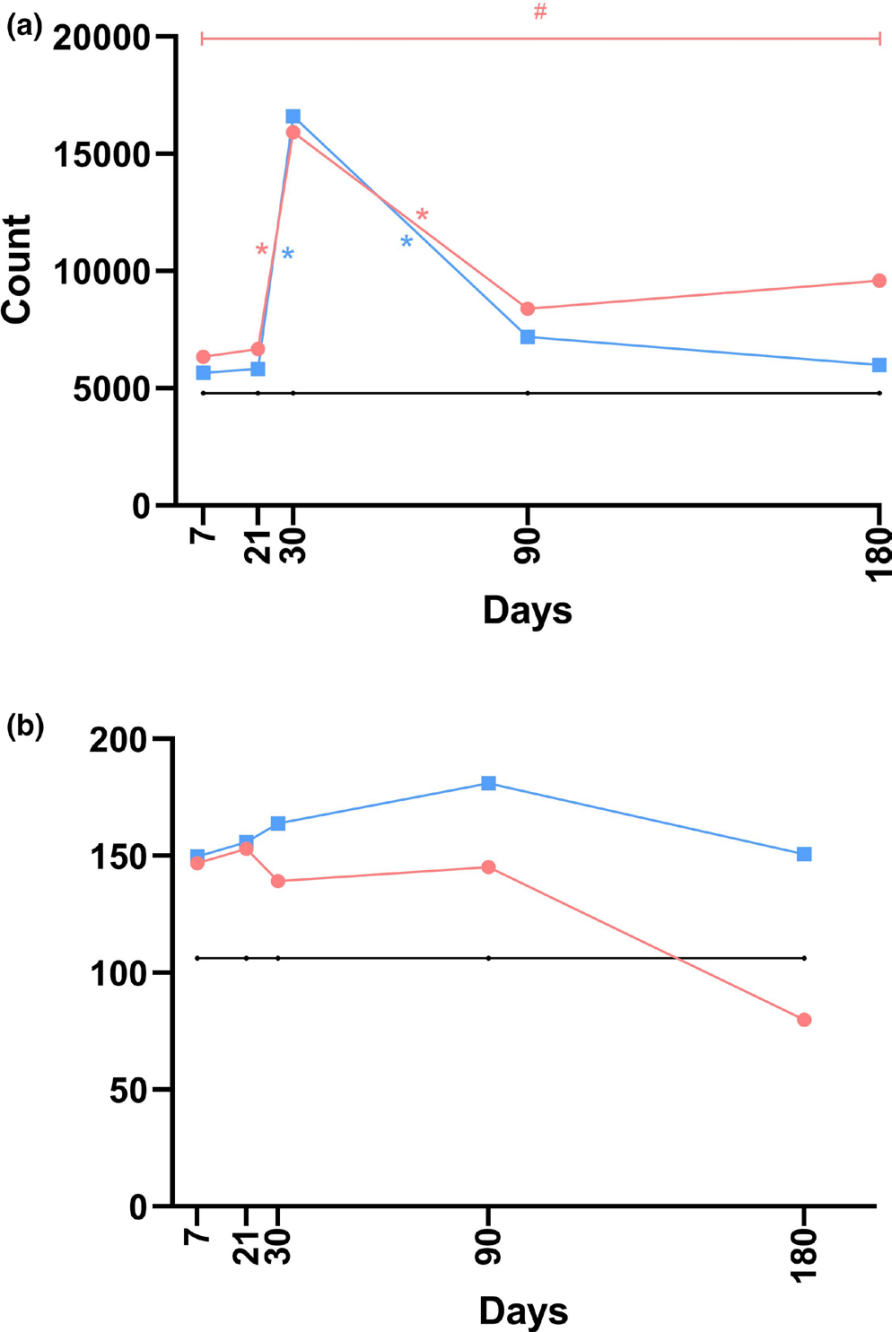
The linear graphs visually display data related to fibrocyte count (a) and collagen density (b) across five distinct time points in the post‐implantation period, constructed from median values. In these graphs, the *black* line represents the control group, the *blue* line indicates the P(LA/CL)‐HA threads, and the *red* line corresponds to the P(LA/CL) threads. The results were considered statistically significant at *p* < 0.05 (* for 0.01 ≤ *p* < 0.05), whereas a *p*‐value ranging from 0.05 to 0.1 (#) suggested a potential trend toward significance, implying that further significance could be achieved with an increased sample size.

The results from the histological analysis, which specifically targeted fibrocytes, are visually illustrated in Figures 6 and S5.

#### 3.3.6 Collagen Density

*P(LA/CL)* vs. *P(LA/CL)‐HA*. The collagen density was significantly greater when P(LA/CL)‐HA threads were used than when P(LA/CL) threads were used.

The results of the histological analysis, which focused specifically on collagen density, are graphically depicted in Figures 6 and S6.

#### 3.3.7 Density of Type I Collagen in the Dermis

*Control* vs. *P(LA/CL)*. The type I collagen density in the dermis, when P(LA/CL) was used, exceeded the control values from day 7 to day 90, with a statistically significant difference observed on day 90 and a trend toward a statistically significant difference noted on day 7. However, there was a sharp decrease on day 180, although this change was not statistically significant.

*Control* vs. *P(LA/CL)‐HA*. The levels of type I collagen in the dermis, when P(LA/CL)‐HA was used, exceeded the control values throughout the entire study period, with statistically significant differences observed on days 30 and 90.

*P(LA/CL)* vs. *P(LA/CL)‐HA*. The density of type I collagen in the dermis, when P(LA/CL)‐HA was used, resulted in higher values on days 7, 30, and 180 than those achieved with P(LA/CL) threads, with the difference being statistically significant on day 180. Conversely, on days 21 and 90, the values for P(LA/CL) exceeded those for P(LA/CL)‐HA, with the difference being statistically significant on day 90.

**FIGURE 7:**
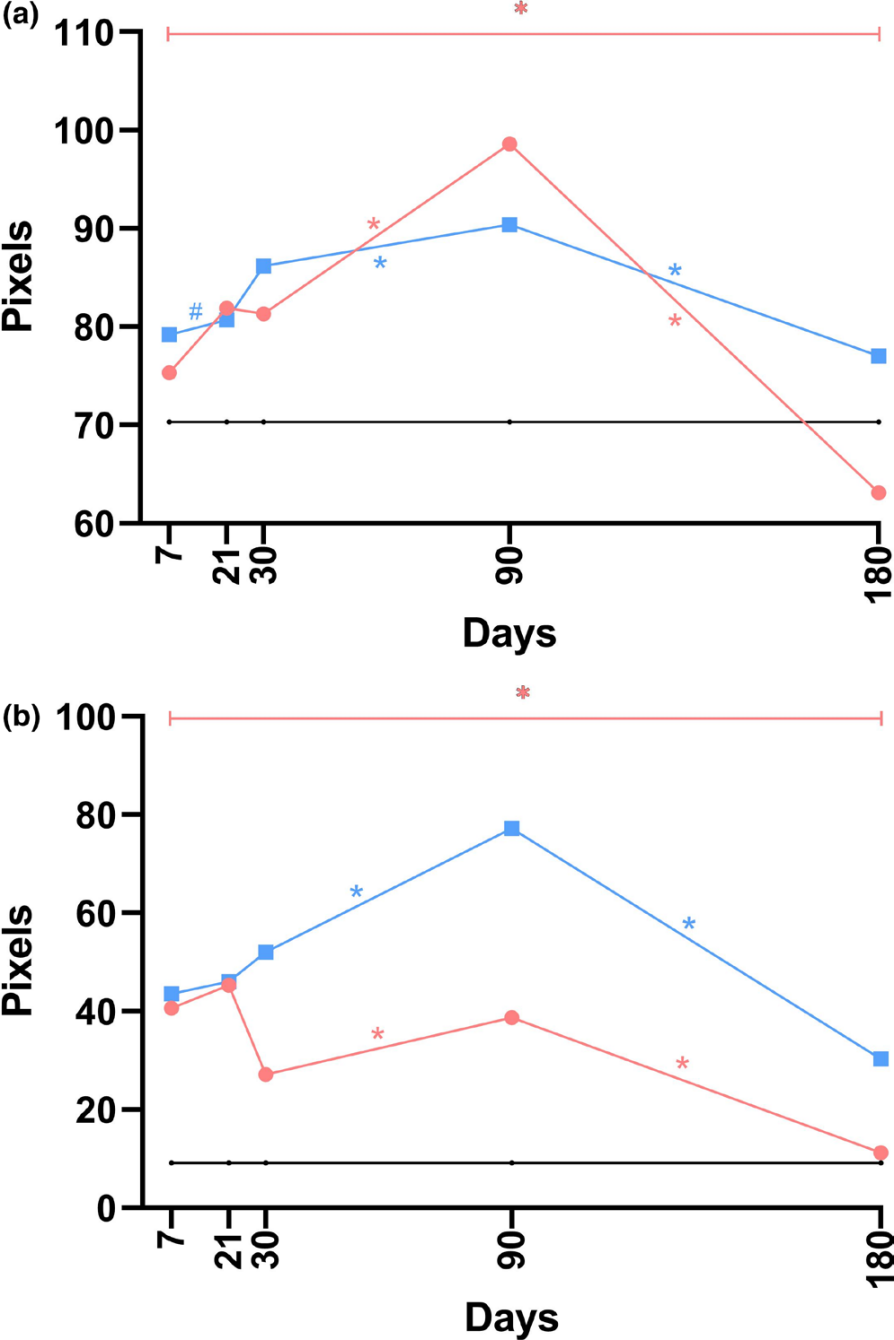
The linear graphs methodically present data concerning the density of type I collagen in the dermis (a) and hypodermis (b) at five separate time points following implantation, using median values for graphical construction. In these visual representations, the *black* line denotes the control group, whereas the *blue* and *red* lines indicate the P(LA/CL)‐HA threads and P(LA/CL) threads, respectively. The results were considered statistically significant at *p* < 0.05 (* for 0.01 ≤ *p* < 0.05), and a tendency toward a significant difference was suggested for 0.05 < *p* < 0.1 (#), indicating that significance might be established with an increased number of samples analyzed.

The data from the histological analyses, which specifically focused on type I collagen density in the dermis, are graphically presented in Figures 7 and S7.

#### 3.3.8 Density of Type I Collagen in the Hypodermis

*Control* vs. *P(LA/CL)*. The type I collagen density in the hypodermis, when P(LA/CL) was used, consistently exceeded the control values throughout the entire study period, with statistically significant differences observed on days 7, 21, 30, and 90.

*Control* vs. *P(LA/CL)‐HA*. Similar to the levels of type I collagen in the dermis, the density in the hypodermis consistently exceeded the control values throughout the entire study period when P(LA/CL)‐HA was used. Statistically significant differences were observed on all days.

*P(LA/CL)* vs. *P(LA/CL)‐HA*. The density of type I collagen in the hypodermis, when P(LA/CL)‐HA was used, exceeded the values observed with P(LA/CL) at every time interval and reached its peak on day 90 before sharply decreasing. In contrast, when P(LA/CL) was used, this indicator peaked on day 21, decreased by day 30, increased again by day 90, and then decreased almost to the control level by day 180. On days 30, 90, and 180, the difference in this parameter with P(LA/CL)‐HA was significantly greater than that observed with P(LA/CL).

The results of the histological analysis, which specifically focused on the density of type I collagen in the hypodermis, are presented in Figures 7 and S8.

#### 3.3.9 Density of Type III Collagen in the Dermis

*Control* vs. *P(LA/CL)*. The type III collagen density in the dermis when P(LA/CL) was used was consistently lower than the control values throughout all days. Statistically significant differences were observed on days 7 and 90, and there was a trend toward a statistically significant difference on day 180.

*Control* vs. *P(LA/CL)‐HA*. The levels of type III collagen in the dermis were consistently lower in the P(LA/CL)‐HA group than in the control group on all days. Statistically significant differences were observed on days 30, 90, and 180.

*P(LA/CL)* vs. *P(LA/CL)‐HA*. The density of type III collagen in the dermis, when P(LA/CL)‐HA was used, exceeded the values observed with P(LA/CL) on days 7 and 90, with the difference being statistically significant on day 90. Conversely, on days 21, 30, and 180, the values for P(LA/CL) were greater than those for P(LA/CL)‐HA; however, the difference was not statistically significant on day 180, and a trend toward a significant difference was noted. Notably, when P(LA/CL)‐HA was used, this indicator steadily decreased over all the days studied. In contrast, with P(LA/CL), it remained relatively unchanged on days 7, 21, and 30, decreased sharply on day 90, but returned to its initial values by day 180.

**FIGURE 8:**
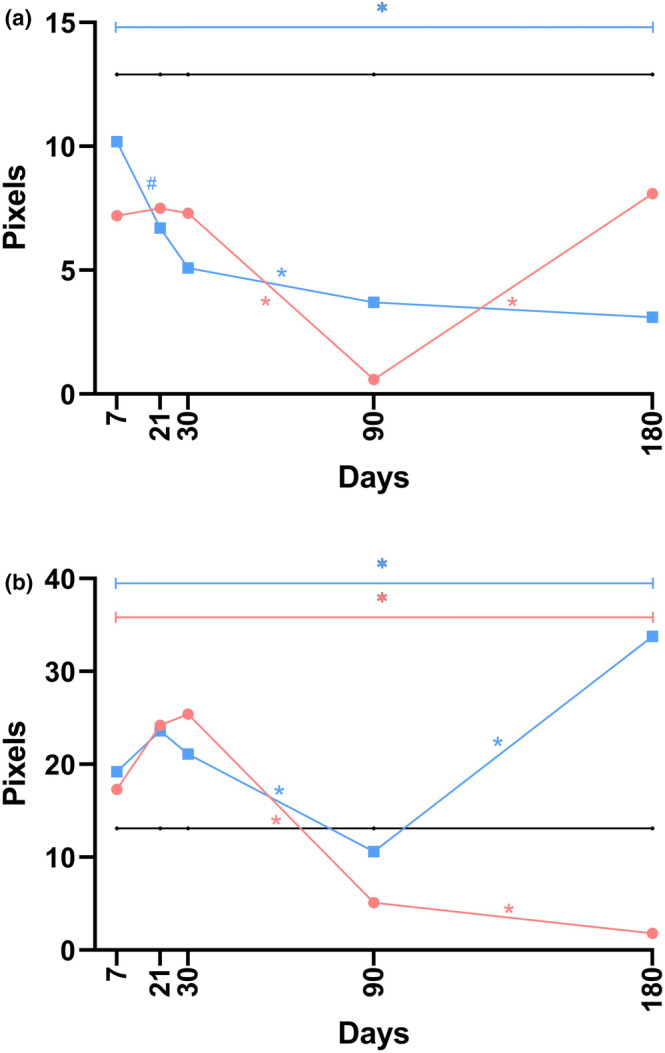
The linear graphs systematically depict data related to the density of type III collagen in the dermis (a) and hypodermis (b) across five distinct time points post‐implantation, constructed using median values. Within these graphical representations, the *black* line represents the control group, whereas the *blue* and *red* lines correspond to the P(LA/CL)‐HA threads and P(LA/CL) threads, respectively. The results with a *p*‐value less than 0.05 were deemed statistically significant (* for 0.01 ≤ *p* < 0.05), and a *p*‐value ranging from 0.05 to 0.1 (#) indicated a potential trend toward significance, suggesting that definitive significance could be reached with a larger dataset.

The results from the histological examination, which specifically targeted the density of type III collagen in the dermis, are graphically depicted in Figures 8 and S9.

#### 3.3.10 Density of Type III Collagen in the Hypodermis

*Control* vs. *P(LA/CL)*. The type III collagen density in the hypodermis, when P(LA/CL) was used, exceeded the control values from day 7 to day 30, with statistically significant differences observed on days 21 and 30. However, by day 90, the values for this parameter had decreased and were significantly lower than the control values.

*Control* vs. *P(LA/CL)‐HA*. The levels of type III collagen in the hypodermis significantly exceeded the control values when P(LA/CL)‐HA was used from day 7 to day 30. By day 90, the values for this parameter had decreased and were significantly lower than the control values. On day 180, the values for this indicator increased again when P(LA/CL)‐HA was used and were significantly greater than the control values.

*P(LA/CL)* vs. *P(LA/CL)‐HA*. The density of type III collagen in the hypodermis, when P(LA/CL)‐HA was used, exceeded the values observed with P(LA/CL) on days 7, 90, and 180, reaching its maximum on day 180. Conversely, when P(LA/CL) was used, this indicator peaked on day 30 and then decreased, reaching its lowest value on day 180. On days 90 and 180, the difference in this parameter with P(LA/CL)‐HA was significantly greater than that with P(LA/CL).

Findings from the histological analysis, specifically concerning the density of type III collagen in the hypodermis, are visually presented in Figures 8 and S10.

#### 3.3.11 Ratio of Type I/III Collagen in the Dermis

*Control* vs. *P(LA/CL)*. The ratio of type I/III collagen in the dermis when P(LA/CL) threads were used was greater than the control value. Statistically significant differences were observed on days 7 and 90.

*Control* vs. *P(LA/CL)‐HA*. The ratio of type I/III collagen in the dermis when P(LA/CL)‐HA threads were used was greater than the control value. Statistically significant differences were observed on days 30, 90, and 180.

*P(LA/CL)* vs. *P(LA/CL)‐HA*. The ratio of type I/III collagen in the dermis, when P(LA/CL) and P(LA/CL)‐HA were used, did not differ on days 7, 21, and 30. However, a sharp increase in this indicator was noted on day 90 for P(LA/CL), resulting in a statistically significant difference compared with P(LA/CL)‐HA. By day 180, this parameter had decreased for P(LA/CL), and the values for P(LA/CL)‐HA had become significantly greater than those for P(LA/CL).

**FIGURE 9:**
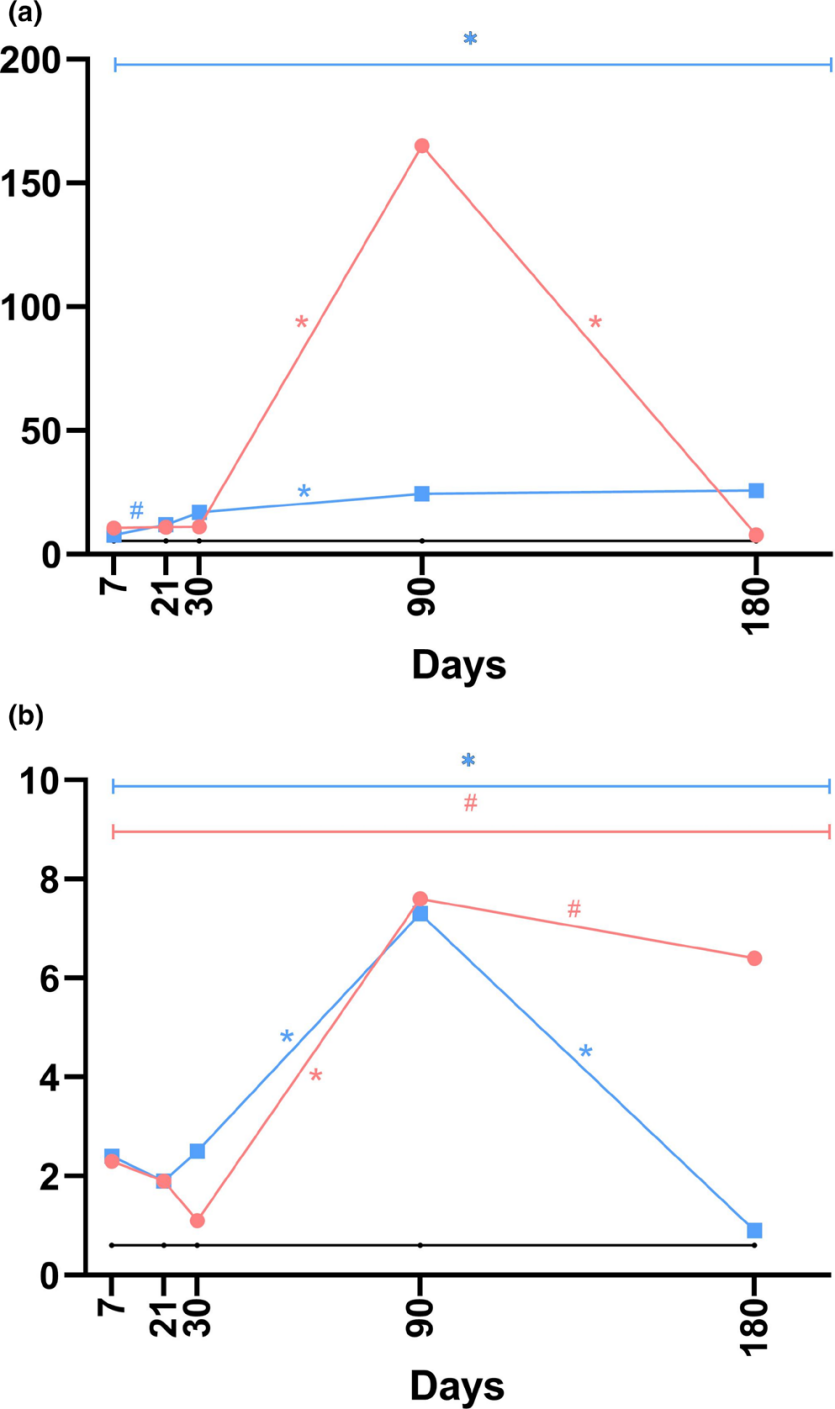
The linear graphs methodically represent data pertaining to the ratio of type I to type III collagen in the dermis (a) and hypodermis (b) at five separate time points following implantation, utilizing median values for the construction. In these visual representations, the *black* line denotes the control group, whereas the *blue* and *red* lines indicate the P(LA/CL)‐HA threads and P(LA/CL) threads, respectively. The results that yielded a *p*‐value of less than 0.05 were considered statistically significant (* for 0.01 ≤ *p* < 0.05), and a *p*‐value ranging from 0.05 to 0.1 (#) suggested a potential trend toward significance, implying that conclusive significance could be achieved with an expanded sample size.

Data from the histological evaluation, which focused on the ratio of type I to type III collagen in the dermis, are graphically illustrated in Figures 9 and S11.

#### 3.3.12 Ratio of Type I/III Collagen in the Hypodermis

*Control* vs. *P(LA/CL)*. The ratio of type I/III collagen in the hypodermis when P(LA/CL) was used exceeded the control values throughout the entire study period. Statistically significant differences were observed on days 7, 21, 90, and 180, whereas a trend toward statistically significant differences was noted on day 30.

*Control* vs. *P(LA/CL)‐HA*. The ratio of type I/III collagen in the hypodermis when PLA‐HA threads were used consistently and significantly exceeded the control values throughout the entire study period.

*P(LA/CL)* vs. *P(LA/CL)‐HA*. The ratio of type I/III collagen in the hypodermis, when both P(LA/CL) and P(LA/CL)‐HA were used, showed almost no difference on days 7 and 21. However, a decrease in this indicator was noted on day 30 for P(LA/CL), whereas for P(LA/CL)‐HA, the parameter increased, resulting in a statistically significant difference between them. On day 90, the ratio of type I/III collagen in the hypodermis sharply increased for both types of threads, but the difference between P(LA/CL) and P(LA/CL)‐HA was not statistically significant. By day 180, this parameter had slightly decreased for P(LA/CL) and remained at the same high level, whereas the values for P(LA/CL)‐HA had significantly decreased, making the difference between P(LA/CL) and P(LA/CL)‐HA on day 180 statistically significant.

The results from the histological analysis, which specifically targeted the ratio of type I to type III collagen in the hypodermis, are visually presented in Figures 9 and S12.

#### 3.3.13 Density of Elastin in the Dermis

*Control* vs. *P(LA/CL)*. The elastin density in the dermis, when P(LA/CL) threads were used, was lower than the control values on days 7 and 21, with the difference being statistically significant on day 21. However, by day 30, it had increased, and the values for P(LA/CL) exceeded those of the control. A trend toward statistically significant differences continued through days 30, 90, and 180.

*Control* vs. *P(LA/CL)‐HA*. The levels of elastin in the dermis, when P(LA/CL)‐HA threads were used, were below the control value on day 7. From days 21 to 180, the values for P(LA/CL)‐HA exceeded those of the control. A trend toward statistically significant differences was noted on days 21 and 30, and by day 180, the difference became statistically significant.

*P(LA/CL)* vs. *P(LA/CL)‐HA*. For the density of elastin in the dermis, a statistically significant difference was observed only on day 21, when the indicator for P(LA/CL) sharply decreased, reaching its lowest value, whereas it increased for P(LA/CL)‐HA. On day 30, as well as on day 180, this parameter for P(LA/CL) exceeded the values for P(LA/CL)‐HA, but the differences were not statistically significant. On day 90, the values of the indicators for both P(LA/CL) and P(LA/CL)‐HA were the same.

**FIGURE 10:**
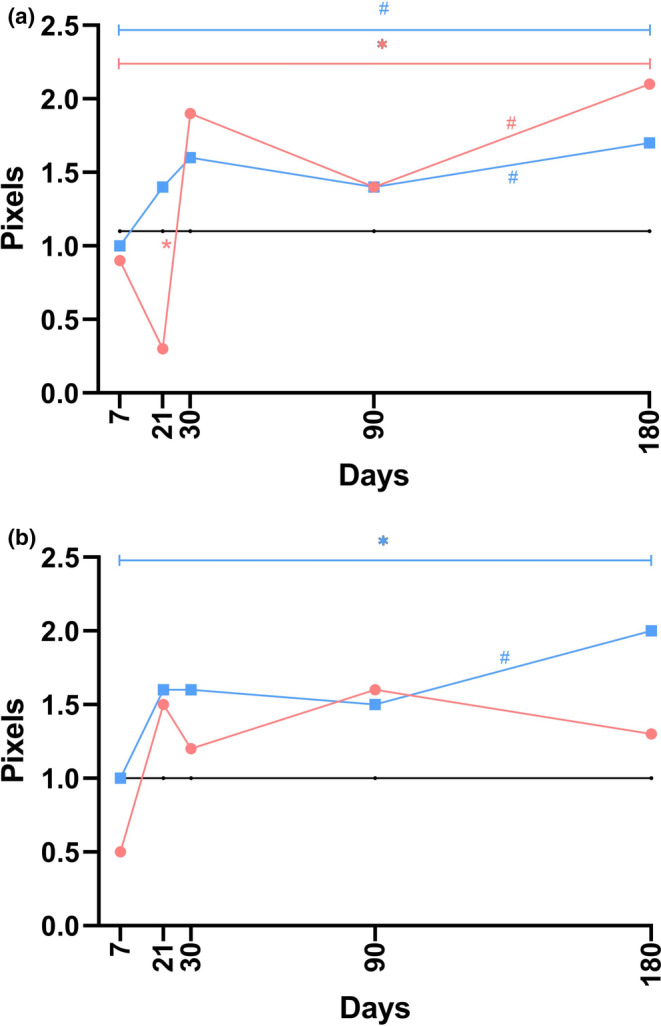
The linear graphs methodically illustrate data concerning the density of elastin in the dermis (a) and hypodermis (b) at five specific time points during the post‐implantation period, constructed from median values. In these visual representations, the *black* line denotes the control group, whereas the *blue* and *red* lines represent the P(LA/CL)‐HA threads and P(LA/CL) threads, respectively. The results were considered statistically significant at *p* < 0.05 (* for 0.01 ≤ *p* < 0.05), whereas a *p*‐value ranging from 0.05 to 0.1 (#) suggested a potential trend toward significance, implying that further significance could be achieved with an increased sample size.

Findings from the histological examination, specifically focusing on elastin levels in the dermis, are graphically depicted in Figures 10 and S13.

#### 3.3.14 Density of Elastin in the Hypodermis

*Control* vs. *P(LA/CL)*. The elastin density in the hypodermis, when P(LA/CL) threads were used, was below the control values on day 7 but increased by day 21 and remained above the control levels until day 180. A trend toward statistically significant differences was observed on days 90 and 180.

*Control* vs. *P(LA/CL)‐HA*. For the P(LA/CL)‐HA threads, the initial density of the elastin in the hypodermis was at the control value on day 7, followed by a rise on day 21, with levels remaining above the control value until day 180. Notably, this indicator increased further by day 180. Patterns of statistically significant differences were also observed on days 90 and 180.

*P(LA/CL)* vs. *P(LA/CL)‐HA*. For the levels of elastin in the hypodermis, no statistically significant differences were observed across all time intervals. On days 7, 21, 30, and 180, the values for P(LA/CL)‐HA exceeded those of P(LA/CL). Conversely, on day 90, the value for P(LA/CL) was slightly greater than that for P(LA/CL)‐HA.

The results from the histological analysis, which specifically targeted the density of elastin in the hypodermis, are visually illustrated in Figures 10 and S14.

## 4 Discussion

### 4.1 Dermal Thickness

On the 7th and 21st days, the dermal thickness associated with the P(LA/CL)‐HA threads was lower than that associated with the P(LA/CL) threads. This initial lower measurement might suggest a slower onset of the tissue response to HA‐enhanced threads. The HA component may modulate the early inflammatory response differently than simple P(LA/CL) threads do. HA is known for its anti‐inflammatory properties and role in maintaining hydration and tissue homeostasis, which might initially moderate the swelling and fibrotic response that contributes to increased thickness [15, 16, 17, 18, 19, 20].

By the 30th day, the dermal thickness measurements of the two thread types were equal. This suggests that any initial delays in response associated with the HA threads catch up as the HA begins to exert its effects on the tissue environment. This equalization indicates that at this time point, the beneficial properties of HA, such as promoting fibroblast activity and enhancing extracellular matrix (ECM) components, begin to manifest more evidently, compensating for the initial slower response [15, 21].

By the 180th day, the dermal thickness in the P(LA/CL)‐HA group was significantly greater than that in the P(LA/CL) group. This significant increase underscores the long‐term benefits of HA in thread form. As HA helps maintain sustained enhancement of tissue hydration and supports continuous collagen synthesis, the tissue structure becomes more robust [16, 18, 19]. The increased dermal thickness could be indicative of a more substantial collagen matrix and a more resilient dermal layer. Additionally, the long‐term presence of HA might also facilitate a more organized deposition of collagen fibers, leading to an overall increase in skin thickness [16, 21].

The data indicate that while the immediate response to P(LA/CL)‐HA threads in the porcine model may be subtler or slower, the long‐term effects are profoundly beneficial, leading to a greater increase in dermal thickness. This finding aligns with HA's known effects on skin physiology, including its ability to enhance tissue repair, modulate inflammatory responses, and support collagen remodeling and maturation [16, 18, 20, 21].

### 4.2 Thickness of the Fibrous Sheath

The findings from the experimental study indicate significant variability in the fibrous sheath thickness surrounding P(LA/CL) threads with and without HA over a period ranging from 7 to 180 days. Early observations at 7, 21, and 90 days post‐implantation revealed that P(LA/CL) threads elicited a thicker fibrous sheath than did P(LA/CL)‐HA threads. This could suggest an initial heightened inflammatory or fibrotic response to P(LA/CL) threads, which may be attributable to the mechanical properties of the threads or their interaction with the surrounding cellular environment. However, this trend reversed at later time points (30 and 180 days), where P(LA/CL)‐HA threads demonstrated a significantly greater thickness of the fibrous sheath.

These observations are intriguing when placed alongside the literature on the biostimulatory effects of hyaluronic acid in tissue engineering [17, 20]. Previous research has emphasized the role of HA in enhancing tissue hydration and facilitating the organization of ECM components, which increases the degree of robust and sustained collagen remodeling [18, 21]. The delayed increase in fibrous sheath thickness associated with HA‐containing threads may reflect a prolonged biostimulatory effect, leading to continuous collagen deposition and potentially a more stable integration of the thread within the treated tissue.

Moreover, the variation in the thickness of the fibrous capsule over time underscores the dynamic nature of the tissue response to different thread compositions. The initial stronger response to P(LA/CL) threads without HA could be indicative of a more aggressive fibrotic reaction, which may be beneficial in applications requiring rapid tissue stabilization. Conversely, the progressive increase in response to P(LA/CL)‐HA threads aligns with applications where long‐term tissue compatibility and fewer inflammatory reactions are desired, such as facial aesthetic procedures where gradual but long‐lasting results are preferable.

These findings from an animal model emphasize the importance of thread composition. P(LA/CL) threads prompt a quick tissue response, potentially beneficial for acute wound healing and reducing early post‐operative complications. The inclusion of HA modulates this response, resulting in gradual, robust integration that is ideal for enhancing long‐term outcomes and the quality of tissue remodeling.

### 4.3 Morphometric Characteristics of Vessels

Our results demonstrated notable variability in the diameter of blood vessels and the relative vascular bed area surrounding the threads, which are critical factors influencing tissue integration and healing. In the case of the vascular diameter and bed area response, initially, the P(LA/CL) threads induced a more pronounced increase in the diameter of the blood vessels than did the controls which was particularly significant on day 21 and trending toward significance on day 180. This enhanced vascular response in the early to midterm stages post‐implantation could be indicative of an acute inflammatory response coupled with the onset of angiogenesis. Interestingly, this response temporarily subdued on day 30, suggesting a possible physiological adjustment or remodeling phase in the vascular architecture.

In contrast, threads incorporated with HA (P(LA/CL)‐HA) consistently presented increases in both vessel diameter and vascular bed area across all time points, with statistical significance achieved by day 180. This sustained vascularization may reflect the well‐documented biostimulatory properties of HA, which are known to facilitate angiogenesis and enhance tissue hydration and integration. The presence of HA appears to modulate the tissue response, potentially smoothing the peaks of the inflammatory response and supporting a more continuous tissue remodeling process.

In the comparison between threads, no significant differences were observed between the two thread types in terms of vascular diameter, except on specific days where the fluctuations noted could reflect minor differences in how each material interacts with the tissue milieu over time. However, the overall similar patterns in the vascular response suggest that the core thread material (P(LA/CL)) fundamentally drives vascular changes, with HA providing a modulatory but not transformative effect on this specific parameter.

Finally, enhanced vascularization around the implant sites could translate to improved clinical outcomes, including better thread integration and more natural‐looking, lasting results from lifting procedures. Moreover, the minimal differences in the vascular response between thread types underscore the potential for HA to enhance but not drastically alter the fundamental properties of P(LA/CL) threads in the case of blood supply modulation.

### 4.4 Fibrocytes

In the case of the fibrocyte response to thread implantation, our findings indicate that both P(LA/CL) and P(LA/CL)‐HA threads effectively stimulate fibrocyte activity compared with that of untreated controls, corroborating the literature that has demonstrated the biostimulatory effects of thread lifting materials on the dermal matrix [22]. Notably, the HA‐enhanced threads presented an earlier onset of significant fibrocyte activity, with statistical significance observed as early as day 21 post‐implantation, compared with day 30 for standard P(LA/CL) threads. This early activation suggests that HA may play a role in accelerating the cellular response, which is crucial for initiating the remodeling processes necessary for effective skin rejuvenation.

For the comparative efficacy of P(LA/CL) vs. P(LA/CL)‐HA, while both thread types presented a peak in fibrocyte numbers by day 30, indicating a robust response to both materials, P(LA/CL)‐HA threads presented a more pronounced peak. This observation could be attributed to the hygroscopic nature of HA, which may enhance moisture retention at the site of implantation, thereby creating a more conducive environment for the cellular activities essential for wound healing and tissue regeneration [19, 21, 23]. The subsequent decline in the number of fibrocytes across both thread types after 30 days likely reflects the transition from an acute inflammatory and proliferative phase to a more mature remodeling phase of wound healing.

In this study, the trend toward a statistically significant difference in fibrocyte counts by day 180 in the P(LA/CL)‐HA thread group compared with the control group underscores the potential for HA‐enhanced biomaterials to sustain fibrocyte activity over prolonged periods. While these results are promising, the translation of findings from an animal model to human clinical practice necessitates careful consideration. In clinical terms, sustained fibrocyte activity may be indicative of enhanced tissue remodeling capabilities, which could lead to more robust and longer‐lasting aesthetic improvements in patients.

### 4.5 Collagenogenesis

Our findings reveal a dynamic interaction between these biomaterials and the host tissue, characterized by varying degrees of collagen synthesis over a period extending up to 180 days post‐implantation.

Compared with those in the control group, both types of threads stimulated a significant increase in type I collagen in the dermis and hypodermis, with P(LA/CL)‐HA threads showing generally superior performance over non‐HA threads. This enhanced response can be attributed to the hyaluronic acid coating, which likely promotes greater biocompatibility and a more sustained fibrotic response [20, 21]. These results are consistent with previous research indicating that hyaluronic acid can enhance cellular activity and tissue integration, thus supporting a more robust and durable collagen network [15, 21].

The differential patterns of collagen synthesis observed between the dermis and hypodermis underscore the role of tissue‐specific responses to biomaterials. While the dermal response highlights the immediate and transient nature of fibrotic activity, which peaks at 90 days, the hypodermal response demonstrates prolonged collagen activity.

Interestingly, the decline in collagen levels on day 180 for P(LA/CL) threads in the dermis may reflect the beginning of material degradation or a resolution of the initial fibrotic response, which does not seem to occur as pronounced with the HA‐enhanced threads. This finding suggests that the integration of HA not only prolongs the bioactive phase but also may contribute to the stabilization of the collagen matrix as the threads begin to degrade, which aligns with studies that have shown the role of HA in maintaining tissue hydration and elasticity [17, 18, 19, 23].

Moreover, the significant differences observed between the thread types on days 30 and 180 in the hypodermis, where P(LA/CL)‐HA threads maintained higher collagen levels, align with the notion that HA‐modified materials might offer better long‐term effects in terms of tissue support and rejuvenation.

In the case of stimulation of type III collagenogenesis, our findings illuminate the complex interplay between thread composition and the biological remodeling of the ECM, particularly regarding the early and late phases of tissue integration.

In the dermis, both P(LA/CL) and P(LA/CL)‐HA threads induced a consistently lower type III collagen response than the control samples did throughout the study period. This observation suggests that the presence of these threads may initially inhibit the typical surge in type III collagen that characterizes the early inflammatory phase of tissue healing. This could be attributed to the mechanical properties of the threads, which might impede the natural proliferative response of fibroblasts to injury. Notably, the addition of hyaluronic acid did not ameliorate this response; rather, it was associated with persistently lower levels of type III collagen. The statistically significant reductions in type III collagen levels with P(LA/CL)‐HA thickening on days 30, 90, and 180 might reflect an accelerated transition to a more mature collagenous matrix, a process potentially facilitated by HA interactions with tissue hydrodynamics and fibroblast functionality.

Conversely, the hypodermal response painted a different picture. Initially, P(LA/CL) threads stimulated an increase in type III collagen levels above control values, peaking between days 21 and 30 before decreasing. This transient enhancement suggests that the threads may temporarily increase collagen synthesis or remodeling in deeper tissue layers before regression to baseline levels or below, as observed by day 90. In contrast, the P(LA/CL)‐HA threads maintained higher Type III collagen levels for a longer duration, notably rebounding by day 180 to levels significantly higher than those of both the control and P(LA/CL) threads. This rebound could indicate a secondary wave of fibroblastic activity or a delayed but sustained enhancement of the collagen matrix owing to the modulatory effects of HA on cellular and extracellular components.

The fluctuating patterns of type III collagen production observed with both thread types in the dermis and hypodermis highlight the complex temporal dynamics of the tissue response to biomaterials. The initial inhibition followed by a late‐stage resurgence (particularly with HA‐enhanced threads) underscores the potential for these materials to serve not only as mechanical scaffolds but also as bioactive platforms that modulate tissue remodeling over extended periods.

Manipulation of the ECM composition, particularly the balance between type I and type III collagen, is critical in aesthetic medicine and tissue engineering. The ratio of type I to type III collagen serves as an important biomarker for the maturation of the ECM and the quality of tissue remodeling following the implantation of bioabsorbable threads.

In our analysis, both P(LA/CL) and P(LA/CL)‐HA threads demonstrated the ability to alter the natural balance of collagen types in the dermis and hypodermis compared with untreated controls. Notably, the presence of these threads increased the ratio of type I to type III collagen, suggesting a shift toward more mature and structurally stable collagen formation. This shift was statistically significant on multiple occasions throughout the 180‐day study period, highlighting the strong influence of thread composition on the tissue response.

In the dermis, the P(LA/CL) threads, in particular, accelerated the maturation process in the ECM, as evidenced by higher ratios of type I/III collagen, especially on days 7 and 90. These findings suggest that the mechanical stimulation and biochemical environment created by P(LA/CL) threads might favor the rapid organization and cross‐linking of collagen fibers. However, this rapid maturation could lead to premature stabilization of the ECM, which might not be desirable in all clinical scenarios.

Conversely, the inclusion of hyaluronic acid in P(LA/CL)‐HA threads appeared to modulate this maturation process. The HA‐enhanced threads showed a smoother and sustained increase in the type I/III collagen ratio, particularly at later stages (day 180), indicating a prolonged period of tissue remodeling. This could be attributed to the hygroscopic properties of HA, which maintain hydration in the tissue microenvironment, potentially facilitating ongoing fibroblast activity and a more gradual deposition of mature collagen fibers [15, 21, 23].

Furthermore, the distinct responses observed between the dermis and hypodermis highlight the importance of considering tissue‐specific dynamics when employing thread‐lifting techniques. The hypodermis, which is typically richer in adipocytes and has a different vascular and fibrous structure than the dermis responds differently over time, underscoring the need for tailored thread‐lifting strategies on the basis of targeted anatomical layers.

Our findings align with and expand upon the current literature, indicating that thread composition can significantly influence tissue healing and remodeling pathways [22]. The differences in collagen type ratios induced by P(LA/CL) versus P(LA/CL)‐HA threads suggest that the choice of thread can be strategically made on the basis of desired clinical outcomes, particularly concerning the longevity and quality of aesthetic improvements.

Finally, our study revealed that both types of threads distinctly altered the balance between type I and type III collagen, highlighting a shift toward a more mature and structurally stable collagen composition, which was particularly evident in the enhanced performance of the P(LA/CL)‐HA threads. These findings underscore the critical role of thread composition in directing tissue responses, suggesting that the incorporation of hyaluronic acid into thread materials not only prolongs the bioactive phase but also potentially stabilizes the collagen matrix during critical periods of degradation and integration. While these findings from an animal model are promising, further validation in human clinical trials is necessary to confirm their clinical efficacy.

### 4.6 Elastogenesis

Our findings revealed a dynamic response in elastin synthesis within the dermis and hypodermis, which varied over time and between thread compositions.

Initially, both thread types presented a decrease in elastin levels in the dermis relative to those of the control samples, which was particularly pronounced on day 7 and persisted until day 21 for P(LA/CL) threads. This initial reduction might be attributed to the mechanical disruption caused by thread insertion or an early inflammatory response, as suggested by Leslie Baumann et al. (2021), who noted that environmental and mechanical stresses could temporarily impair elastin integrity and synthesis in skin tissues [24].

Interestingly, from day 30 onward, both P(LA/CL) and P(LA/CL)‐HA threads induced a notable increase in dermal elastin levels above control values, with statistically significant enhancements observed in the long term. This increase aligns with previous findings that interventions stimulating elastin production can lead to substantial improvements in skin structure and function [24]. The presence of hyaluronic acid in P(LA/CL)‐HA threads likely contributed to the more pronounced and earlier increase in elastin synthesis, underscoring the role of hyaluronic acid in enhancing the cellular activities that support elastogenesis.

The sustained increase in elastin levels in the hypodermis up to day 180 for both thread types suggests robust activation of elastogenic pathways, which might involve the recruitment and activation of fibroblasts capable of synthesizing elastin as part of the tissue remodeling process [25].

Moreover, the differential response observed between the thread types on day 21 in the dermis, where P(LA/CL) threads decreased and P(LA/CL)‐HA threads increased elastin, highlights the potential modifying effects of hyaluronic acid on the inflammatory and remodeling processes initiated by thread insertion. Such differential impacts might be indicative of the varying kinetics of biodegradation and bioactivity between the two thread formulations, as hypothesized in the literature discussing bioabsorbable polymers by Vincent Wong (2021) [10].

In conclusion, our study provides evidence that both P(LA/CL) and P(LA/CL)‐HA threads can effectively stimulate elastogenesis in skin tissues in an animal model, with enhanced effects observed with hyaluronic acid –augmented threads.

## 5 Conclusion

Our study extensively evaluated the dynamic interaction between two types of bioabsorbable threads, P(LA/CL) and P(LA/CL)‐HA, and their impact on skin remodeling over a period of 180 days in a porcine model. The findings reveal a complex interplay between thread composition and tissue response, significantly enriching our understanding of the material properties and their potential optimization for future applications.

Compared with standard P(LA/CL) threads, the inclusion of hyaluronic acid in P(LA/CL)‐HA threads modulated fibrotic and inflammatory responses. Initially, HA appeared to reduce acute inflammatory responses, likely because of its known anti‐inflammatory and hydrating properties. Over time, this modulation led to more sustained and organized collagen deposition, as indicated by increased dermal thickness and enhanced fibrous sheath structure in the later stages of the experiment.

This study also highlighted thread‐induced changes in vascular architecture. Enhanced vascularization around the thread implant sites, particularly in the HA‐enhanced threads, suggests the potential for improved tissue integration.

At the cellular level, both types of threads stimulate fibrocyte activity and collagen production, with HA threads showing earlier onset and sustained activity. Furthermore, both thread types stimulated elastogenesis, with the HA‐augmented threads showing a more pronounced effect on increasing elastin synthesis.

In conclusion, our results underscore the importance of thread composition in influencing tissue response and remodeling outcomes in a porcine model. The addition of hyaluronic acid to P(LA/CL) threads not only modulates inflammation and fibrosis but also enhances long‐term remodeling processes. These findings suggest potential for future applications; however, further studies in clinical settings are essential to validate these results and explore their implications for therapeutic and aesthetic outcomes in humans.

## Author Contributions

Conceptualization, P.B., G.S., and D.N.; methodology, P.B. and D.N.; software, P.B.; validation, D.N.; formal analysis, P.B.; investigation, P.B. and D.N.; resources, G.S.; data curation, P.B.; writing – original draft preparation, P.B.; writing – review and editing, D.N.; visualization, P.B.; supervision, D.N.; project administration, P.B.; funding acquisition, G.S. All authors have read and agreed to the published version of the manuscript.

## Ethics Statement

This investigation was conducted in strict accordance with the protocols approved by the Local Ethics Committee (LEC) of Preclinical Research Center LLC (Penza, Russian Federation). The research design received formal approval from the LEC, documented under protocol number 5–2023, issued on August 23, 2023. This study adheres to the ethical standards and international guidelines for the humane treatment of laboratory animals. Moreover, it conforms to the international standards delineated in ISO 10993‐1:2018, “Biological evaluation of medical devices—Part 1: Evaluation and testing within a risk management process,” as well as ISO 10993‐2:2022, “Biological evaluation of medical devices—Part 2: Animal welfare requirements,” ensuring comprehensive compliance with established ethical and procedural norms for biomedical research.

## Conflicts of Interest

P.B., serving as an independent researcher, was affiliated with the University of Palermo (Palermo, Italy). D.N. occupied the position of Deputy Director General for Research and Development at the Russian Office of APTOS LLC (Moscow, Russia). The other contributors affirm that the study was executed without any commercial or financial relationships that might be interpreted as potential conflicts of interest. It is also disclosed that the institutional doctoral fund from the University of Palermo (Palermo, Italy) facilitated the logistical support for P.B. Furthermore, this investigation was financed by a grant from APTOS LLC (Tbilisi, Georgia), which addressed the project's financial requirements, with G.S. managing these funds in alignment with the project's budgetary allocations. The funding body had no role in the design, data collection, analysis, interpretation of the study, the writing of this manuscript, or the decision to submit it for publication.

## Supporting information


**Data S1.** Supplementary Figures and Tables.

## Data Availability

Data supporting the findings of this study are available from the corresponding author upon reasonable request. The data are not publicly available due to privacy concerns related to the private nature of the data. The Supporting Information contain descriptive statistics, which underpin the analyses in this study.
