# Supplementary material for: Efficacy of Lifting Threads Composed of Poly(L‐Lactide‐Co‐ε‐Caprolactone) Copolymers Coated With Hyaluronic Acid: A Long‐Term Study on Biorevitalizing Properties in Skin Remodeling
Source: J Cosmet Dermatol. 2025 Mar 12;24(3):e70077. doi: 10.1111/jocd.70077 (PMC11898156; doi:10.1111/jocd.70077)
Supplement: Supplementary file 1 — Data S1. Supplementary Figures and Tables. [file JOCD-24-e70077-s001.pdf]

Supplementary materials for

Efficacy of Lifting Threads Composed of Poly(L-lactide-co-ε-caprolactone) Copolymers Coated with Hyaluronic Acid: A Long-Term Study on Biorevitalizing Properties in Skin Remodelling.

This file includes:

1. Supplementary Figures and Figure Legends S1 to S14.

2. Supplementary Tables S1 to S3.

1. Supplementary Figures and Figure Legends

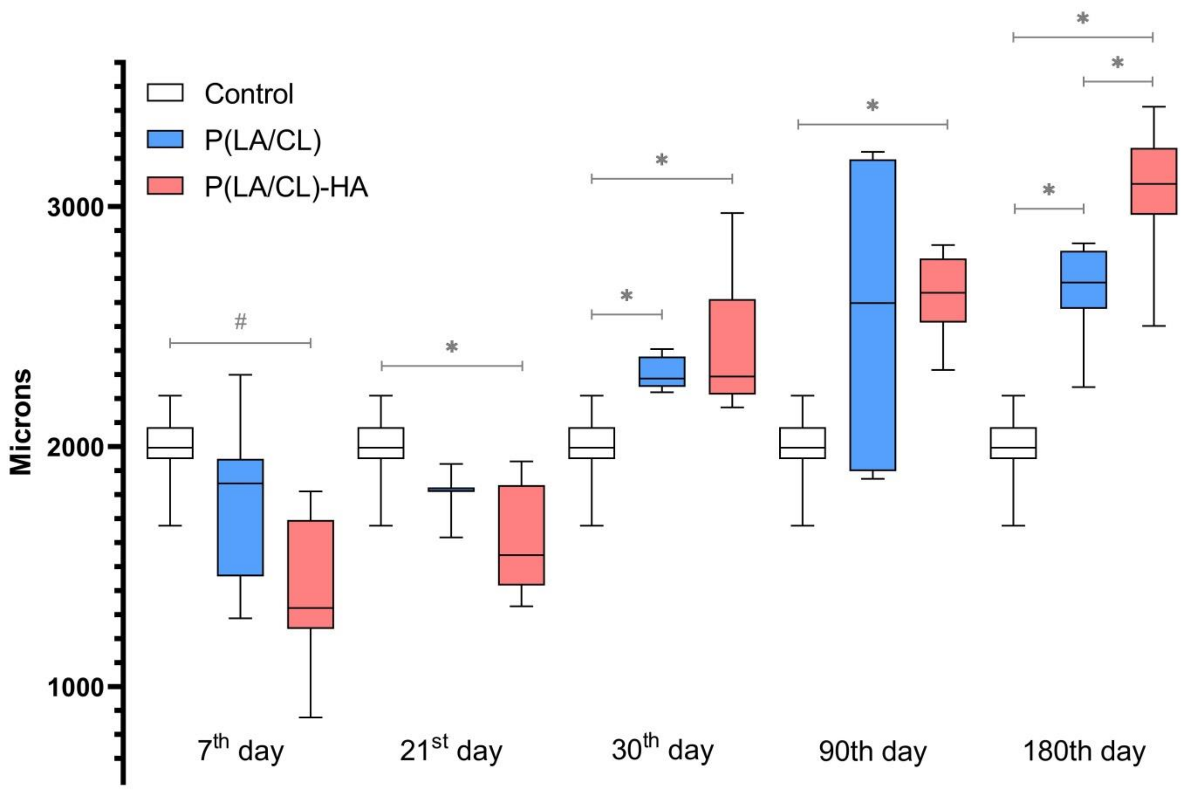

Supplementary Figure S1. A series of box plots displays data on dermal thickness for three groups – namely, the control, P(LA/CL), and P(LA/CL)-HA groups – at five time points during the postimplantation examination. The box plots illustrate a five-number summary: minimum, first quartile (Q1), median, third quartile (Q3), and maximum in microns. These plots provide insights into the variability and distribution of dermal thickness within each group over time, effectively capturing the

central tendency and dispersion of the data. The results that reached a *p*-value of less than 0.05 were deemed statistically significant (* denoting $0.01 \le p < 0.05$).

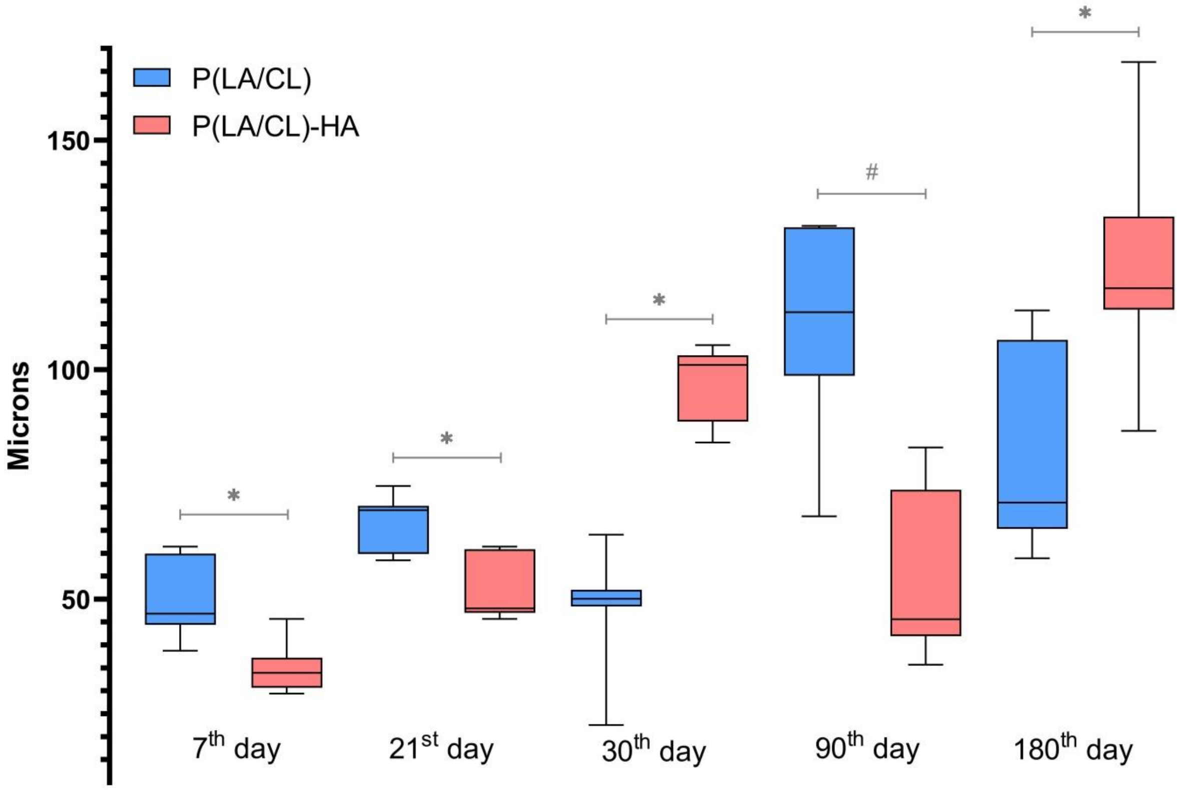

Supplementary Figure S2. A series of box plots graphically presents data on the thickness of the fibrous sheath for three distinct groups – P(LA/CL) and P(LA/CL)-HA – across five time points during the postimplantation period. These box plots render a five-number summary: minimum, first quartile (Q1), median, third quartile (Q3), and maximum, measured in microns. These visual representations offer a comprehensive view of the variability and distribution of fibrous sheath thickness within each group over time, succinctly capturing the central tendencies and data dispersion. The results were considered statistically significant at $p < 0.05$ (* for $0.01 \le p < 0.05$), and a tendency toward a significant difference was suggested for $0.05 < p < 0.1$ (#), indicating that significance might be established with an increased number of samples analysed.

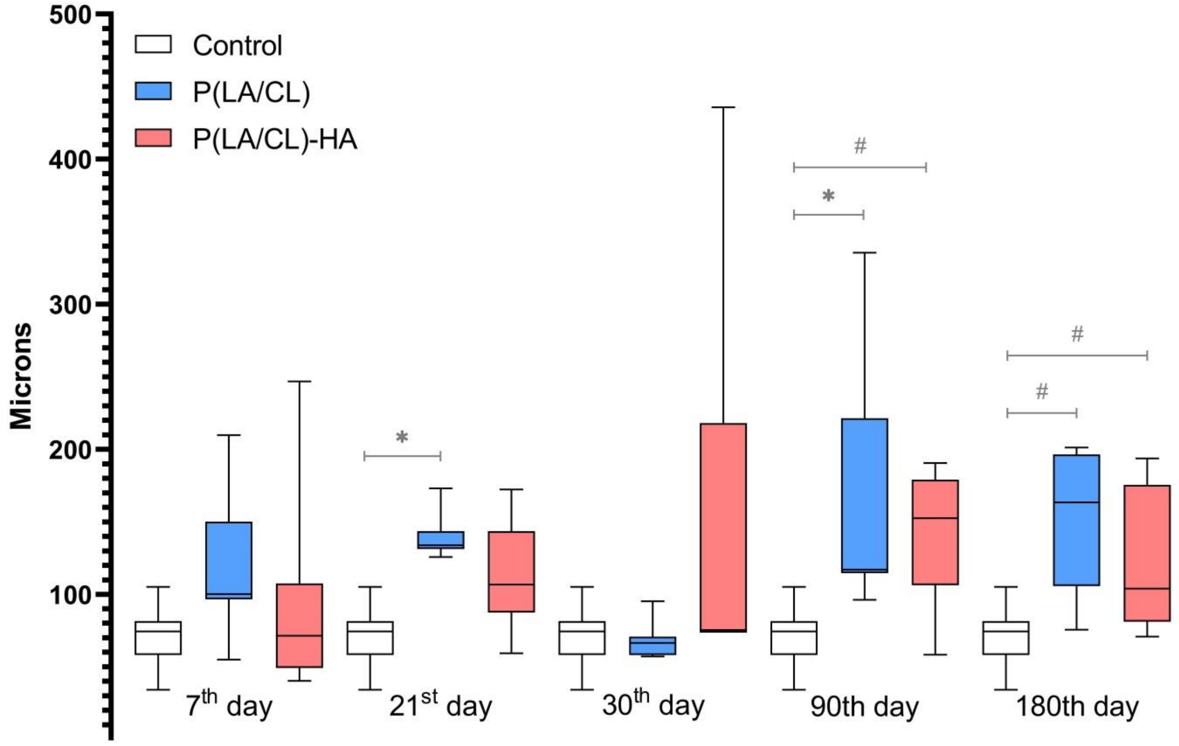

Supplementary Figure S3. A series of box plots visually depicts data on the diameter of blood vessels for three distinct groups: the control, P(LA/CL), and P(LA/CL)-HA groups at five time points during the postimplantation period. These plots provide a five-point summary: minimum, first quartile (Q1), median, third quartile (Q3), and maximum, all measured in microns. This graphical representation affords a detailed analysis of the variability and distribution of blood vessel diameter within each group over time, effectively summarizing the central tendencies and dispersion of the data. The results were considered statistically significant at $p < 0.05$ (* for $0.01 \leq p < 0.05$), whereas a *p*-value ranging from 0.05 to 0.1 (#) suggested a potential trend toward significance, implying that further significance could be achieved with an increased sample size.

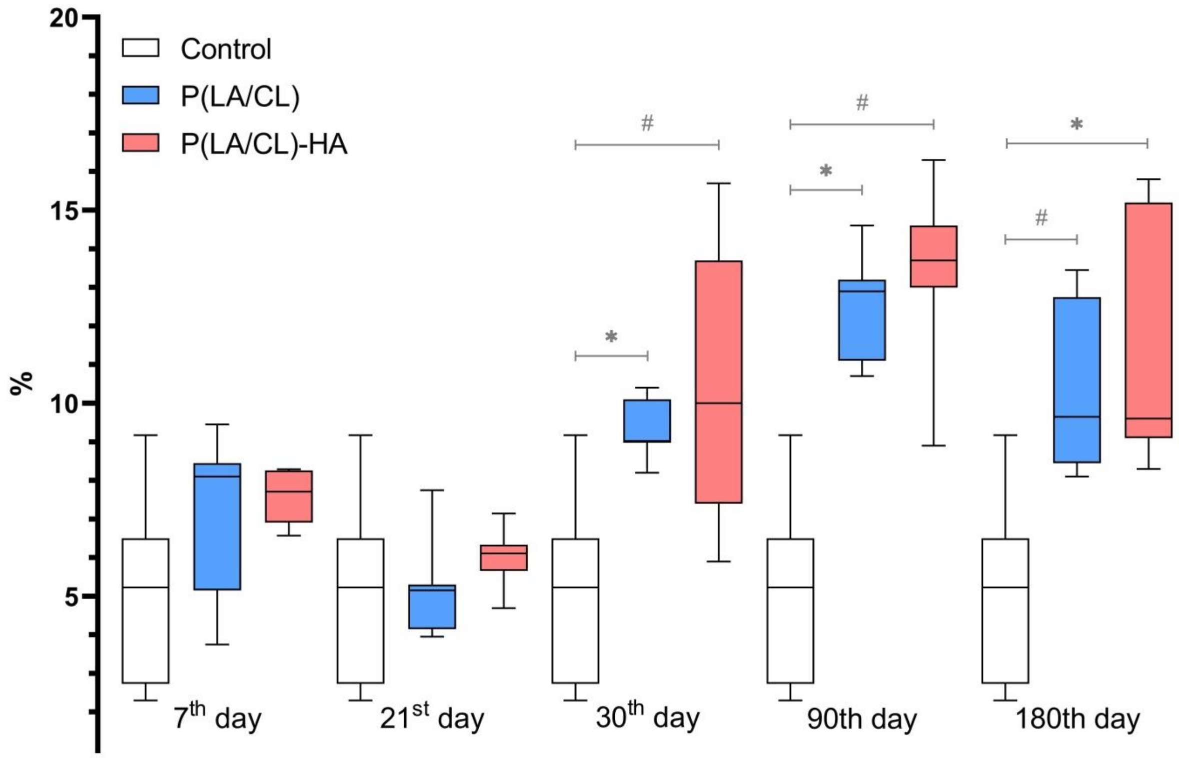

Supplementary Figure S4. A series of box plots visually represents data concerning the relative vascular bed area for three distinct groups – control, P(LA/CL), and P(LA/CL)-HA – across five time points during the postimplantation period. These plots provide a summary of five numbers: minimum, first quartile (Q1), median, third quartile (Q3), and maximum, all of which are quantified in %. This graphical portrayal provides an in-depth examination of the variability and distribution of the relative vascular bed area within each group over time, succinctly encapsulating the central tendencies and variability of the data. The results were considered statistically significant at $p < 0.05$ (* for $0.01 \leq p < 0.05$), and a tendency toward a significant difference was suggested for $0.05 < p < 0.1$ (#), indicating that significance might be established with an increased number of samples analysed.

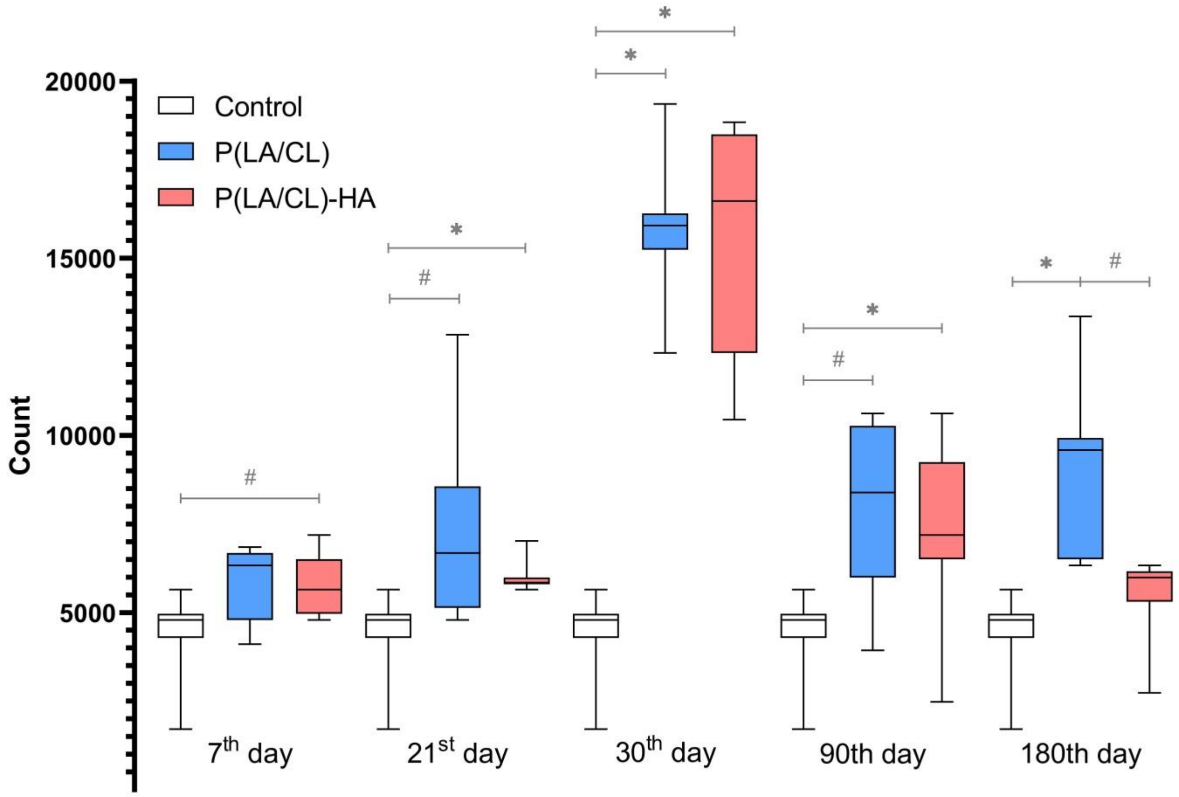

Supplementary Figure S5. A series of box plots graphically illustrates data pertaining to fibrocyte counts for three distinct groups, namely, the control, P(LA/CL), and P(LA/CL)-HA groups, at five time points during the postimplantation period. These plots provide a five-number summary: minimum, first quartile (Q1), median, third quartile (Q3), and maximum, all expressed in counts. This visual representation offers a detailed analysis of the variability and distribution of fibrocyte counts within each group over time, effectively summarizing the central tendencies and dispersion of the data. The results with a *p*-value less than 0.05 were deemed statistically significant (* $0.01 \leq p < 0.05$), and a *p*-value ranging from 0.05 to 0.1 (#) indicated a potential trend toward significance, suggesting that definitive significance could be reached with a larger dataset.

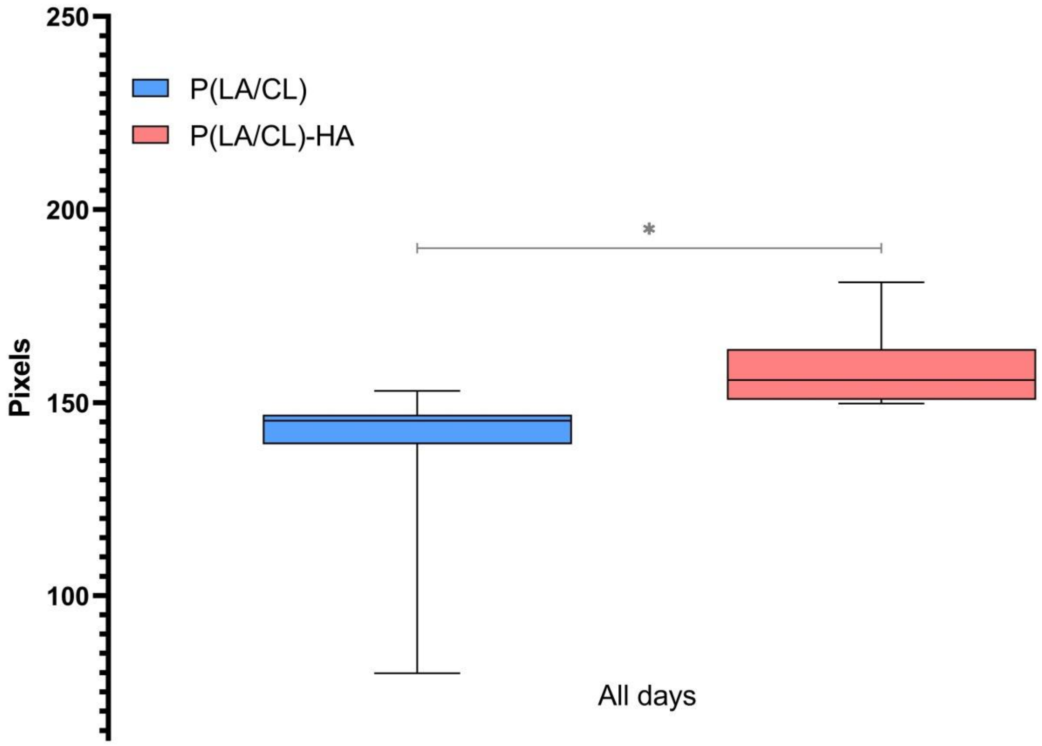

Supplementary Figure S6. A series of box plots methodically illustrates data on collagen density for two distinct groups:, P(LA/CL) and P(LA/CL)-HA, across five time points during the postimplantation period. These plots provide a comprehensive five-number summary: minimum, first quartile (Q1), median, third quartile (Q3), and maximum, each quantified in pixels. This graphical representation provides an exhaustive analysis of the variability and distribution of collagen density within each group over time, effectively delineating the central tendencies and dispersion of the data. The results that achieved a *p*-value of less than 0.05 were considered statistically significant (* for $0.01 \leq p < 0.05$).

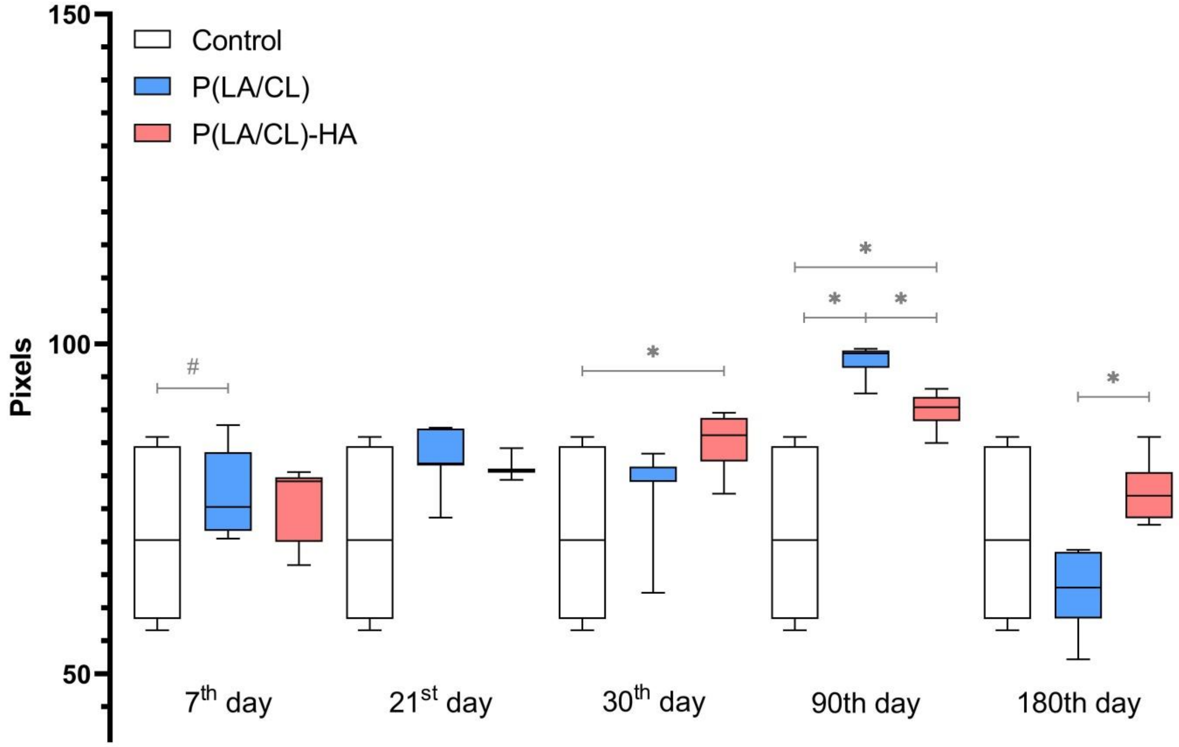

Supplementary Figure S7. A series of box plots methodically presents data concerning the density of type I collagen in the dermis for three distinct groups, namely, the control, P(LA/CL), and P(LA/CL)-HA groups, across five time points during the postimplantation period. These plots provide a summary of five numbers: minimum, first quartile (Q1), median, third quartile (Q3), and maximum, all of which are quantified in pixels. This graphical depiction provides an exhaustive analysis of the variability and distribution of type I collagen density in the dermis within each group over time, effectively capturing the central tendencies and variability of the data. The results that yielded a *p*-value of less than 0.05 were considered statistically significant (* for $0.01 \leq p < 0.05$), and a *p*-value ranging from 0.05 to 0.1 (#) suggested a potential trend toward significance, implying that conclusive significance could be achieved with an expanded sample size.

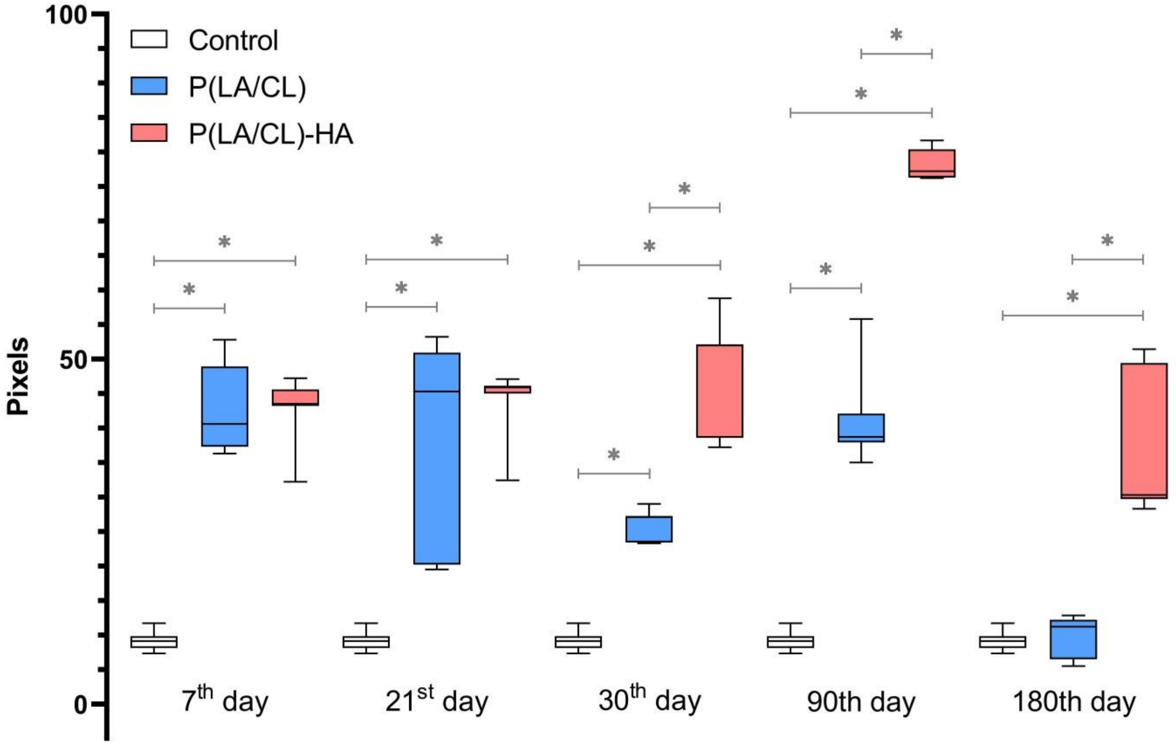

Supplementary Figure S8. A series of box plots were generated to visualize data related to the density of type I collagen in the hypodermis for three distinct groups: the control, P(LA/CL), and P(LA/CL)-HA groups at five time points during the postimplantation period. These plots offer a five-number summary: minimum, first quartile (Q1), median, third quartile (Q3), and maximum, each measured in pixels. This visual representation provides a comprehensive analysis of the variability and distribution of type I collagen density in the hypodermis for each group over time, effectively summarizing the central tendencies and variations in the data. The results were considered statistically significant at $p < 0.05$ (* for $0.01 \leq p < 0.05$).

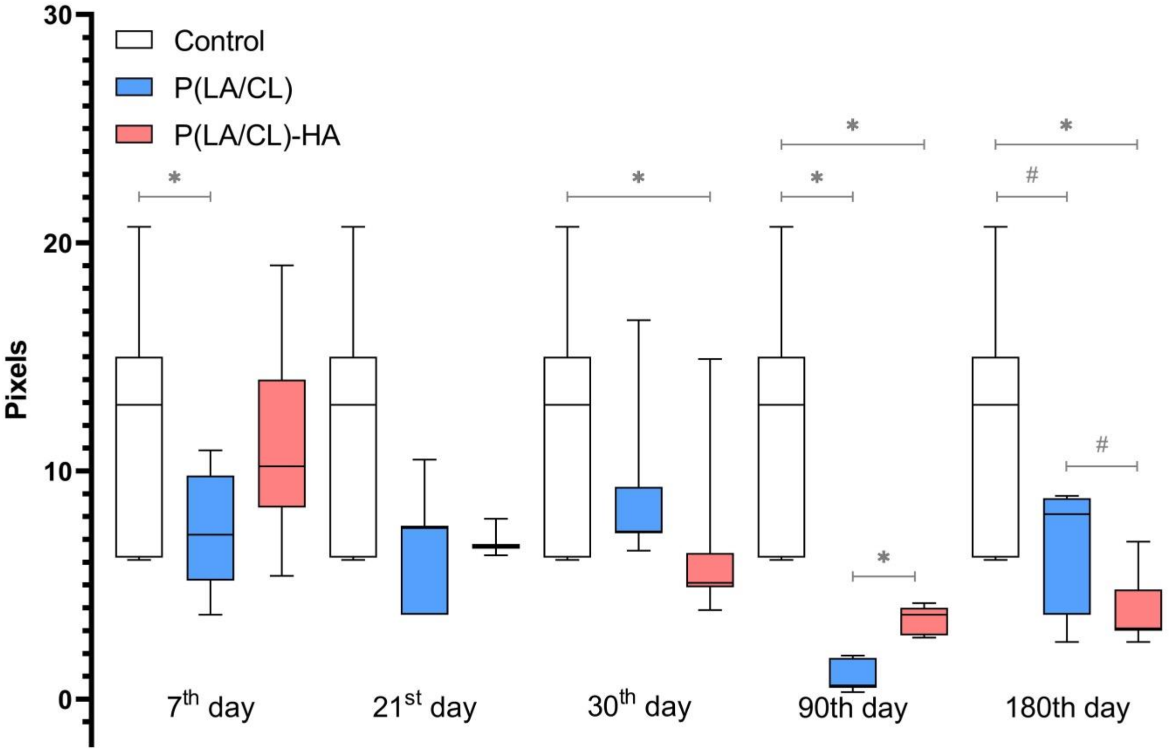

Supplementary Figure S9. A series of box plots rigorously displays data pertaining to the density of type III collagen in the dermis for three distinct groups: the control, P(LA/CL), and P(LA/CL)-HA groups at five sequential time points during the postimplantation period. These plots provide a detailed five-number summary: minimum, first quartile (Q1), median, third quartile (Q3), and maximum, all of which are quantified in pixels. This visual representation offers a thorough analysis of the variability and distribution of type III collagen density in the dermis across each group over time, efficiently delineating the central tendencies and fluctuations in the data. The results were considered statistically significant at $p < 0.05$ (* for $0.01 \leq p < 0.05$), whereas a *p*-value ranging from 0.05 to 0.1 (#) suggested a potential trend toward significance, implying that further significance could be achieved with an increased sample size.

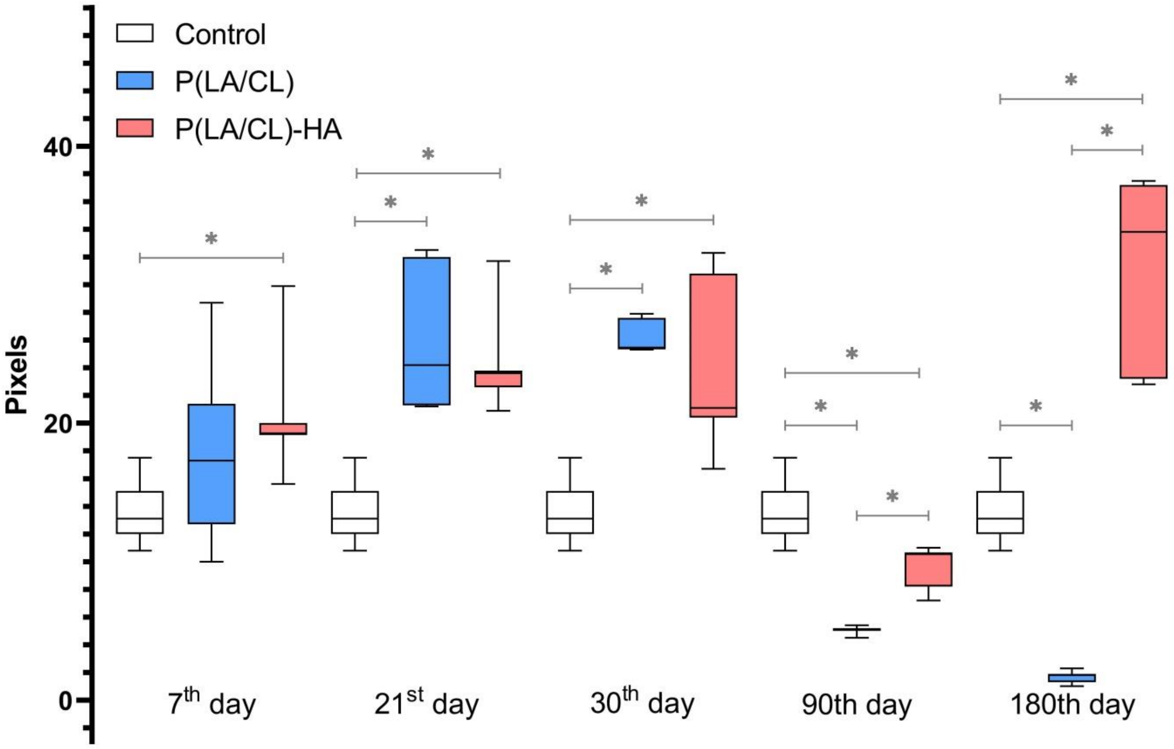

Supplementary Figure S10. A series of box plots methodically illustrates data on the density of type III collagen in the hypodermis for three distinct groups: the control, P(LA/CL), and P(LA/CL)-HA groups, which were captured at five distinct time points during the postimplantation period. These plots generate a five-point summary: minimum, first quartile (Q1), median, third quartile (Q3), and maximum, all of which are quantified in pixels. This graphical illustration provides a detailed examination of the variability and distribution of type III collagen density in the hypodermis across each group over time, effectively encapsulating the central tendencies and range of the data. The results were considered statistically significant at $p < 0.05$ (* for $0.01 \leq p < 0.05$).

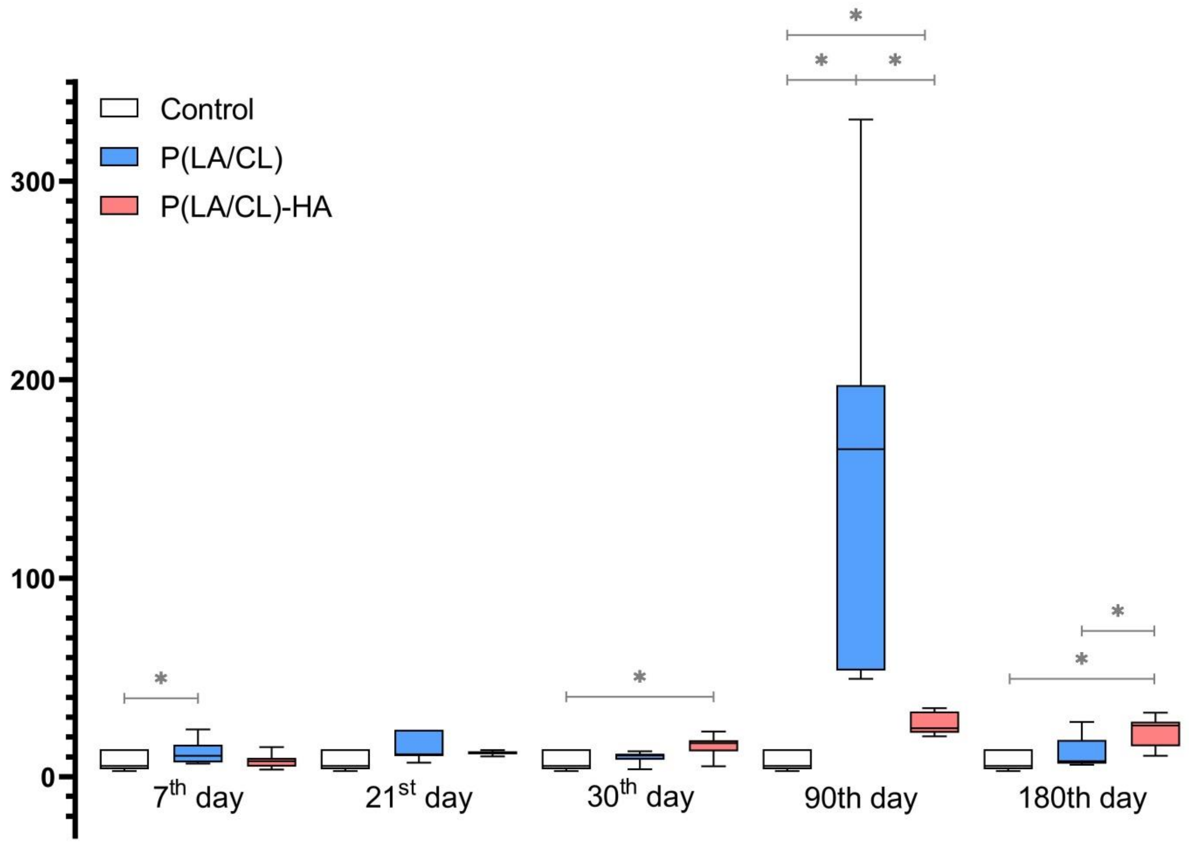

Supplementary Figure S11. A series of box plots rigorously delineates data concerning the ratio of type I to type III collagen in the dermis for three distinct groups, namely, the control, P(LA/CL), and P(LA/CL)-HA groups, recorded at five time points during the postimplantation period. These plots present a comprehensive five-number summary: minimum, first quartile (Q1), median, third quartile (Q3), and maximum. This graphical depiction meticulously analyses the variability and distribution of the type I/III collagen ratio in the dermis among each group over time, effectively capturing the central tendencies and variability of the data. The results with a *p*-value less than 0.05 were deemed statistically significant (* for $0.01 \leq p < 0.05$).

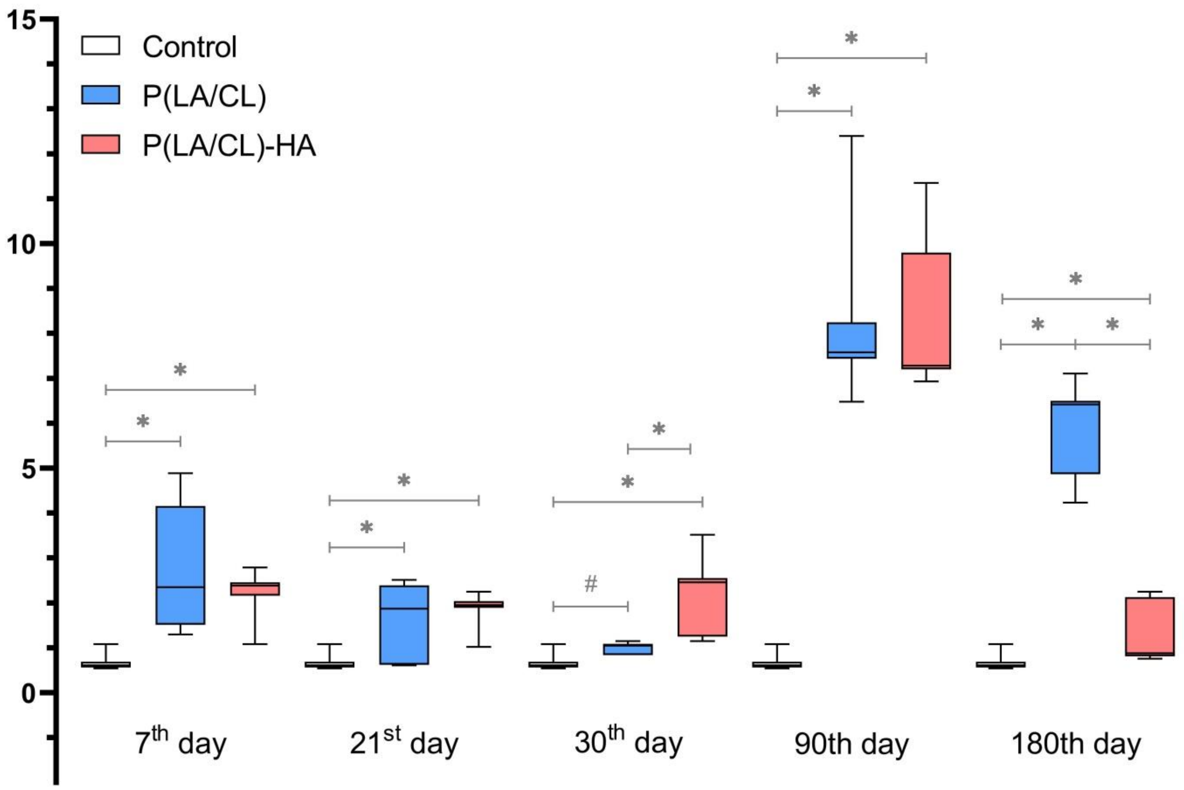

Supplementary Figure S12. A series of box plots were generated to visualize the ratios of type I to type III collagen in the hypodermis across three distinct groups: the control, P(LA/CL), and P(LA/CL)-HA groups at five sequential time points during the postimplantation period. These plots provide a detailed five-number summary: minimum, first quartile (Q1), median, third quartile (Q3), and maximum. This graphical representation offers an exhaustive examination of the variability and distribution of the type I/III collagen ratio in the hypodermis for each group over time, effectively encapsulating the central tendencies and dispersion of the data. The results were considered statistically significant at $p < 0.05$ (* for $0.01 \leq p < 0.05$), and a tendency toward a significant difference was suggested for $0.05 < p < 0.1$ (#), indicating that significance might be established with an increased number of samples analysed.

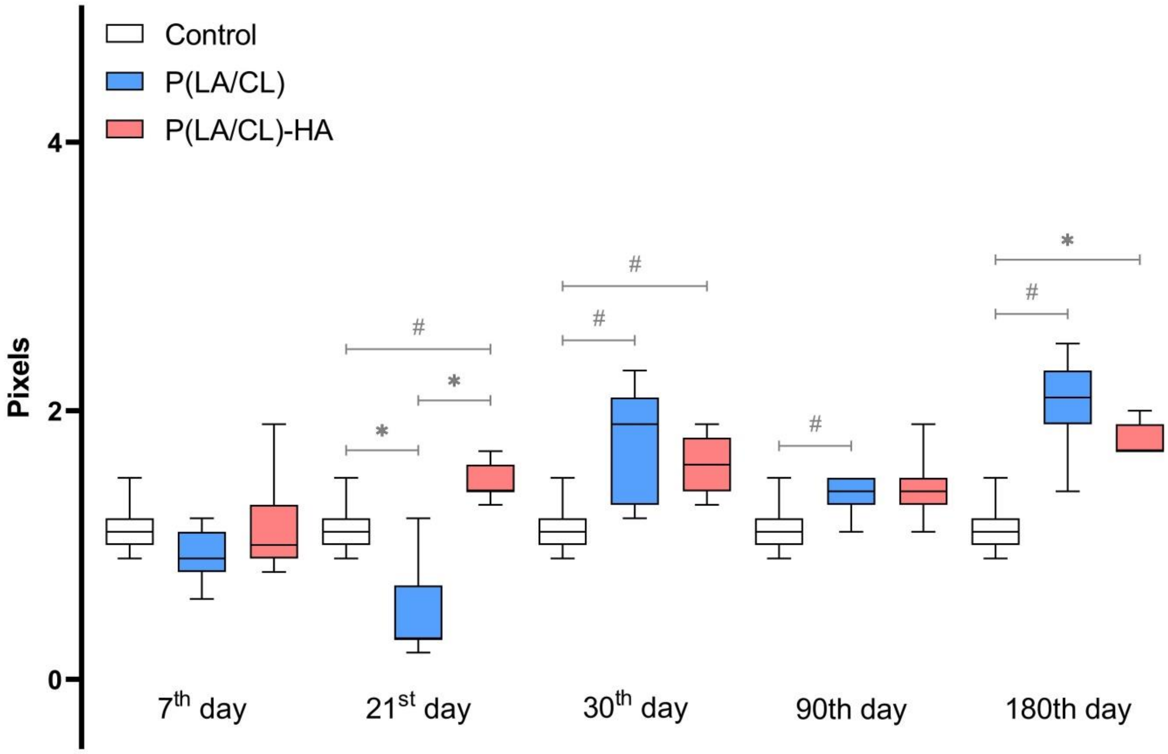

Supplementary Figure S13. A series of box plots methodically presents data concerning the density of elastin in the dermis for three distinct groups: the control, P(LA/CL), and P(LA/CL)-HA groups, which were captured at five consecutive time points during the postimplantation period. These plots provide a comprehensive five-number summary: minimum, first quartile (Q1), median, third quartile (Q3), and maximum, each measured in pixels. This graphical depiction provides an in-depth examination of the variability and distribution of elastin density in the dermis for each group over time, effectively summarizing the central tendencies and variability of the data. The results were considered statistically significant at $p < 0.05$ (* for $0.01 \leq p < 0.05$), whereas a p-value ranging from 0.05 to 0.1 (#) suggested a potential trend toward significance, implying that further significance could be achieved with an increased sample size.

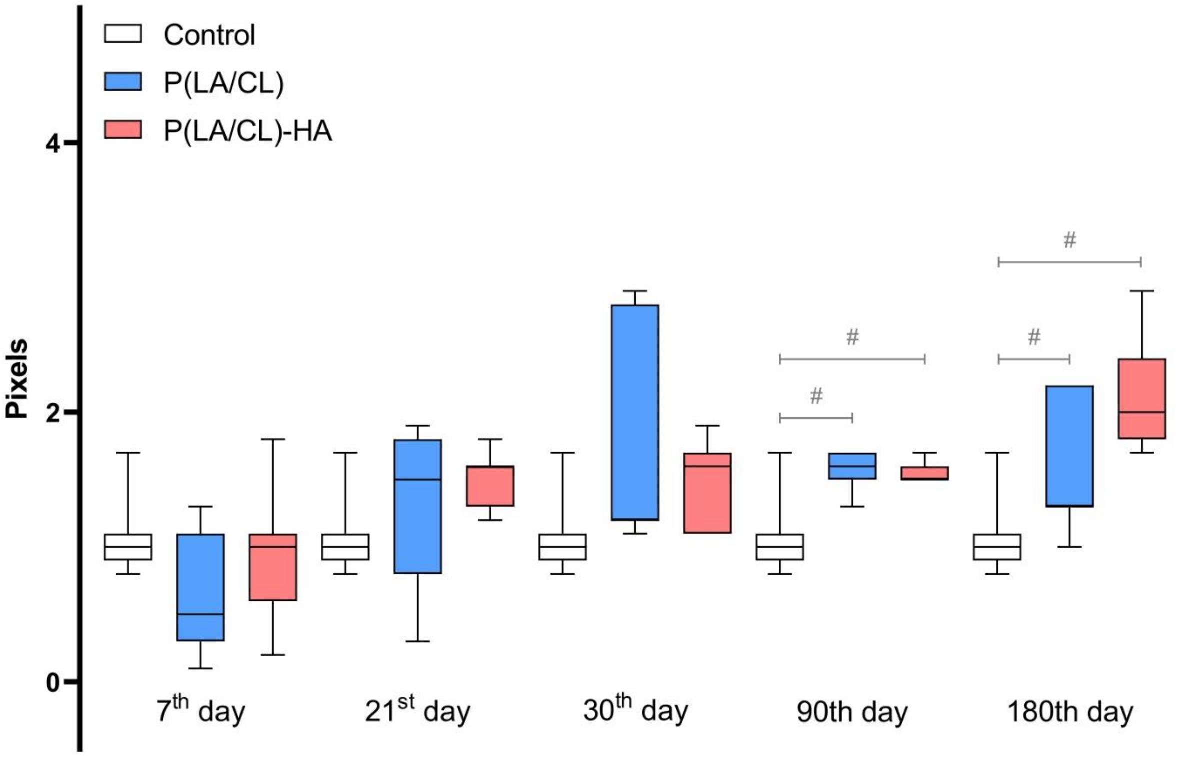

Supplementary Figure S14. A series of box plots visually depicts data on the density of elastin in the hypodermis for three distinct groups: the control, P(LA/CL), and P(LA/CL)-HA groups at five time points during the postimplantation period. These plots provide a five-number summary: minimum, first quartile (Q1), median, third quartile (Q3), and maximum, all measured in pixels. This graphical representation affords a detailed analysis of the variability and distribution of the density of elastin in the hypodermis within each group over time, effectively summarizing the central tendencies and dispersion of the data. The results were considered statistically significant at $p < 0.05$ (* for $0.01 \le p < 0.05$), whereas a *p*-value ranging from 0.05 to 0.1 (#) suggested a potential trend toward significance, implying that further significance could be achieved with an increased sample size.

2. Supplementary tables.

Supplementary Table S1. Descriptive Statistics for P(LA/CL) on Each Studied Day and Across All Days Combined.

Days	M±SD	Me [25%;75%]	Min	Max
**Dermal Thickness, microns**				
All days	2213,48±100,80	2247,12 [1846,62;2574,10]	1284,74	3228,39

Days	M±SD	Me [25%;75%]	Min	Max
7th day	1767,68±180,31	1846,62 [1458,76;1949,18]	1284,74	2299,12
21st day	1801,08±49,71	1814,68 [1811,65;1829,92]	1621,47	1927,69
30th day	2307,93±35,35	2283,86 [2248,69;2374,98]	2225,86	2406,25
90th day	2557,13±298,03	2597,79 [1897,22;3197,22]	1865,01	3228,39
180th day	2633,57±108,21	2683,68 [2574,10;2816,01]	2247,12	2846,95
**Thickness of the Fibrous Sheath, microns**				
All days	71,09±5,70	64,03 [52,01;74,63]	22,58	131,31
7th day	50,26±4,46	46,80 [44,38;59,94]	38,74	61,42
21st day	66,53±3,15	69,37 [59,87;70,32]	58,44	74,63
30th day	47,42±6,79	50,08 [48,39;52,01]	22,58	64,03
90th day	108,31±11,78	112,53 [98,69;130,99]	68,05	131,31
180th day	82,93±11,14	71,04 [65,32;106,50]	58,87	112,90
**Diameter of Blood Vessels, microns**				
All days	131,72±13,03	116,89 [95,26;163,25]	54,93	335,55
7th day	122,30±26,56	100,21 [96,52;150,10]	54,93	209,76
21st day	141,44±8,40	133,91 [131,16;143,58]	125,62	172,95
30th day	69,55±6,92	66,50 [58,15;70,75]	57,07	95,26
90th day	176,91±45,35	116,89 [114,55;221,34]	96,24	335,55
180th day	148,42±24,96	163,25 [105,67;196,38]	75,51	201,27
**Relative Vascular Bed Area, %**				
All days	8,91±0,62	9,00 [7,75;10,70]	3,75	14,60
7th day	6,98±1,08	8,10 [5,15;8,45]	3,75	9,45
21st day	5,26±0,68	5,15 [4,15;5,30]	3,95	7,75
30th day	9,34±0,40	9,00 [9,00;10,10]	8,20	10,40
90th day	12,50±0,72	12,90 [11,10;13,20]	10,70	14,60
180th day	10,48±1,11	9,65 [8,45;12,75]	8,10	13,45
**Fibrocytes, count**				
All days	9232,92±857,29	8390,00 [6336,00;12329,00]	3938,00	19349,00
7th day	5753,60±548,36	6336,00 [4795,00;6678,00]	4110,00	6849,00
21st day	7602,80±1469,50	6678,00 [5137,00;8562,00]	4795,00	12842,00
30th day	15822,00±1122,23	15925,00 [15240,00;16267,00]	12329,00	19349,00
90th day	7842,20±1276,13	8390,00 [5993,00;10274,00]	3938,00	10616,00
180th day	9144,00±1292,26	9589,00 [6507,00;9932,00]	6336,00	13356,00
**Collagen Density, pixels**				
All days	132,86±13,42	145,32 [139,22;146,86]	79,90	153,00
**Density of Type I Collagen in the Dermis, pixels**				
All days	79,43±2,59	81,40 [70,50;87,30]	52,20	99,30
7th day	77,76±3,38	75,30 [71,70;83,60]	70,50	87,70

Days	M±SD	Me [25%;75%]	Min	Max
21st day	82,34±2,49	81,90 [81,60;87,20]	73,70	87,30
30th day	77,50±3,86	81,30 [79,10;81,40]	62,30	83,40
90th day	97,36±1,09	98,60 [96,40;99,00]	93,50	99,30
180th day	62,20±3,15	63,10 [58,40;68,50]	52,20	68,80
**Density of Type I Collagen in the Hypodermis, pixels**				
All days	31,71±3,06	35,00 [20,20;42,10]	5,50	55,80
7th day	43,18±3,27	40,60 [37,30;48,90]	36,30	52,80
21st day	37,82±7,45	45,30 [20,20;50,90]	19,50	53,20
30th day	26,00±1,13	27,10 [23,40;27,20]	23,30	29,00
90th day	41,90±3,65	38,70 [37,90;42,10]	35,00	55,80
180th day	9,64±1,52	11,20 [6,50;12,20]	5,50	12,80
**Density of Type III Collagen in the Dermis, pixels**				
All days	6,16±0,79	7,20 [3,70;8,80]	0,30	16,60
7th day	7,36±1,35	7,20 [5,20;9,80]	3,70	10,90
21st day	6,60±1,30	7,50 [3,70;7,60]	3,70	10,50
30th day	9,40±1,86	7,30 [7,30;9,30]	6,50	16,60
90th day	1,02±0,34	0,60 [0,50;1,80]	0,30	1,90
180th day	6,40±1,37	8,10 [3,70;8,80]	2,50	8,90
**Density of Type III Collagen in the Hypodermis, pixels**				
All days	15,56±2,27	17,30 [5,00;25,40]	1,00	32,50
7th day	18,56±3,48	17,30 [12,70;24,10]	10,00	28,70
21st day	26,24±2,51	24,20 [21,30;32,00]	21,20	32,50
30th day	26,32±0,59	25,40 [25,40;27,60]	25,30	27,90
90th day	5,04±0,15	5,10 [5,00;5,20]	4,50	5,40
180th day	1,66±0,23	1,80 [1,30;1,90]	1,00	2,30
**Ratio of Type I/III Collagen in the Dermis**				
All days	41,98±15,33	11,15 [7,79;23,71]	3,75	331,00
7th day	12,80±3,20	10,46 [7,19;16,08]	6,58	23,71
21st day	15,17±3,50	10,88 [10,78;23,57]	7,02	23,59
30th day	9,48±1,59	11,14 [8,51;11,15]	3,75	12,83
90th day	159,19±52,08	165,00 [53,56;197,20]	49,21	331,00
180th day	13,26±4,24	7,79 [6,56;18,51]	5,93	27,52
**Ratio of Type I/III Collagen in the Hypodermis**				
All days	3,94±0,63	2,51 [1,15;6,48]	0,61	12,40
7th day	2,84±0,72	2,35 [1,51;4,16]	1,30	4,89
21st day	1,60±0,42	1,87 [0,62;2,39]	0,61	2,51
30th day	0,99±0,06	1,07 [0,84;1,07]	0,84	1,15
90th day	8,43±1,03	7,58 [7,44;8,25]	6,48	12,40

Days	M±SD	Me [25%;75%]	Min	Max
180th day	5,83±0,54	6,42 [4,87;6,50]	4,23	7,11
**Density of Elastin in the Dermis, pixels**				
All days	1,32±0,13	1,30 [0,90;1,90]	0,20	2,50
7th day	0,92±0,11	0,90 [0,80;1,10]	0,60	1,20
21st day	0,54±0,19	0,30 [0,30;0,70]	0,20	1,20
30th day	1,76±0,22	1,90 [1,30;2,10]	1,20	2,30
90th day	1,36±0,07	1,40 [1,30;1,50]	1,10	1,50
180th day	2,04±0,19	2,10 [1,90;2,30]	1,40	2,50
**Density of Elastin in the Hypoermis, pixels**				
All days	1,37±0,14	1,30 [1,00;1,70]	0,10	2,90
7th day	0,66±0,23	0,50 [0,30;1,10]	0,10	1,30
21st day	1,26±0,31	1,50 [0,80;1,80]	0,30	1,90
30th day	1,78±0,44	1,20 [1,10;2,80]	0,90	2,90
90th day	1,56±0,07	1,60 [1,50;1,70]	1,30	1,70
180th day	1,60±0,25	1,30 [1,30;2,20]	1,00	2,20

Supplementary Table S2. Descriptive Statistics for P(LA/CL)-HA on Each Studied Day and Across All Days Combined.

Days	M±SD	Me [25%;75%]	Min	Max
**Dermal Thickness, microns**				
All days	2224,19±139,66	2291,30 [1694,34;2783,01]	871,18	3415,64
7th day	1389,06±168,33	1326,79 [1240,45;1694,34]	871,18	1812,54
21st day	1615,90±117,35	1547,48 [1421,23;1838,80]	1334,04	1937,94
30th day	2451,81±151,96	2291,30 [2217,14;2613,92]	2163,65	2973,04
90th day	2619,65±93,86	2640,25 [2516,76;2783,01]	2319,11	2839,12
180th day	3044,56±154,96	3094,64 [2965,29;3244,64]	2502,57	3415,64
**Thickness of the Fibrous Sheath, microns**				
All days	72,81±7,32	61,42 [45,62;101,04]	29,43	167,06
7th day	35,39±2,91	33,91 [30,69;37,24]	29,43	45,68
21st day	52,58±3,51	47,96 [47,02;60,86]	45,65	61,42
30th day	96,48±4,22	101,04 [88,71;103,15]	84,15	105,33
90th day	56,03±9,40	45,62 [41,94;73,86]	35,70	83,03
180th day	123,58±13,21	117,73 [113,09;133,36]	86,68	167,06
**Diameter of Blood Vessels, microns**				
All days	130,89±17,17	106,23 [73,63;175,50]	40,32	435,79
7th day	103,01±37,74	71,42 [49,24;107,38]	40,32	246,71
21st day	113,82±20,01	106,61 [87,42;143,58]	59,35	172,16

Days	M±SD	Me [25%;75%]	Min	Max
30th day	175,29±70,83	75,41 [73,63;217,98]	73,63	435,79
90th day	137,33±24,48	152,47 [106,23;179,03]	58,44	190,49
180th day	124,98±25,06	103,88 [81,22;175,50]	70,75	193,55
**Relative Vascular Bed Area, %**				
All days	9,80±0,75	8,30 [6,91;13,70]	4,69	16,30
7th day	7,55±0,35	7,71 [6,91;8,26]	6,57	8,29
21st day	5,99±0,40	6,11 [5,66;6,34]	4,69	7,14
30th day	10,54±1,85	10,00 [7,40;13,70]	5,90	15,70
90th day	13,30±1,23	13,70 [13,00;14,60]	8,90	16,30
180th day	11,60±1,61	9,60 [9,10;15,20]	8,30	15,80
**Fibrocytes, count**				
All days	8068,60±853,27	6336,00 [5651,00;9247,00]	2740,00	18836,00
7th day	5822,20±456,23	5651,00 [4966,00;6507,00]	4795,00	7192,00
21st day	6061,80±245,82	5822,00 [5822,00;5993,00]	5651,00	7021,00
30th day	15342,60±1685,28	16610,00 [12329,00;18493,00]	10445,00	18836,00
90th day	7808,20±934,13	7192,00 [6507,00;9247,00]	5479,00	10616,00
180th day	5308,20±665,33	5993,00 [5308,00;6164,00]	2740,00	6336,00
**Collagen Density, pixels**				
All days	160,27±5,78	155,86 [150,72;163,86]	149,78	181,14
**Density of Type I Collagen in the Dermis, pixels**				
All days	81,80±1,35	80,70 [79,20;86,20]	66,50	93,20
7th day	75,26±2,89	79,20 [70,20;79,80]	66,50	80,60
21st day	81,18±0,81	80,70 [80,50;81,10]	79,40	84,20
30th day	84,82±2,28	86,20 [82,20;88,80]	77,30	89,60
90th day	89,78±1,45	90,40 [88,30;92,00]	85,00	93,20
180th day	77,94±2,44	77,00 [73,60;80,60]	72,60	85,90
**Density of Type I Collagen in the Hypodermis, pixels**				
All days	49,91±3,29	46,00 [38,60;52,10]	28,30	81,70
7th day	42,34±2,64	43,50 [43,20;45,60]	32,20	47,20
21st day	43,30±2,75	46,00 [45,00;46,00]	32,40	47,10
30th day	47,74±4,21	52,00 [38,60;52,10]	37,20	58,80
90th day	78,36±1,13	77,20 [76,30;80,40]	76,20	81,70
180th day	37,82±5,16	30,30 [29,70;49,40]	28,30	51,40
**Density of Type III Collagen in the Dermis, pixels**				
All days	6,57±0,82	5,40 [3,90;6,90]	2,50	19,00
7th day	11,40±2,36	10,20 [8,40;14,00]	5,40	19,00
21st day	6,86±0,27	6,70 [6,60;6,80]	6,30	7,90
30th day	7,04±2,00	5,10 [4,90;6,40]	3,90	14,90

Days	M±SD	Me [25%;75%]	Min	Max
90th day	3,48±0,31	3,70 [2,80;4,00]	2,70	4,20
180th day	4,06±0,81	3,10 [3,00;4,80]	2,50	6,90
**Density of Type III Collagen in the Hypodermis, pixels**				
All days	22,00±1,75	21,10 [16,70;29,90]	7,20	37,50
7th day	20,78±2,40	19,20 [19,20;20,00]	15,60	29,90
21st day	24,52±1,87	23,60 [22,60;23,80]	20,90	31,70
30th day	24,26±3,08	21,10 [20,40;30,80]	16,70	32,30
90th day	9,52±0,76	10,60 [8,20;10,60]	7,20	11,00
180th day	30,90±3,29	33,80 [23,20;37,20]	22,80	37,50
**Ratio of Type I/III Collagen in the Dermis**				
All days	16,87±1,79	14,93 [10,52;22,77]	3,50	34,52
7th day	8,14±1,99	7,82 [5,01;9,43]	3,50	14,93
21st day	11,91±0,51	11,93 [11,85;12,23]	10,19	13,37
30th day	15,20±2,96	16,90 [12,84;18,29]	5,19	22,77
90th day	26,83±2,89	24,43 [22,08;32,86]	20,24	34,52
180th day	22,29±4,04	25,67 [15,33;27,71]	10,52	32,24
**Ratio of Type I/III Collagen in the Hypoermis**				
All days	3,21±0,58	2,25 [1,25;2,79]	0,76	11,35
7th day	2,17±0,29	2,38 [2,16;2,46]	1,08	2,79
21st day	1,83±0,21	1,95 [1,89;2,04]	1,02	2,25
30th day	2,19±0,44	2,46 [1,25;2,55]	1,15	3,52
90th day	8,51±0,88	7,28 [7,20;9,80]	6,93	11,35
180th day	1,37±0,34	0,88 [0,81;2,13]	0,76	2,25
**Density of Elastin in the Dermis, pixels**				
All days	1,50±0,07	1,50 [1,30;1,70]	0,80	2,00
7th day	1,18±0,20	1,00 [0,90;1,30]	0,80	1,90
21st day	1,48±0,07	1,40 [1,40;1,60]	1,30	1,70
30th day	1,60±0,11	1,60 [1,40;1,80]	1,30	1,90
90th day	1,44±0,13	1,40 [1,30;1,50]	1,10	1,90
180th day	1,80±0,06	1,70 [1,70;1,90]	1,70	2,00
**Density of Elastin in the Hypodermis, pixels**				
All days	1,53±0,11	1,60 [1,20;1,80]	0,20	2,90
7th day	0,94±0,27	1,00 [0,60;1,10]	0,20	1,80
21st day	1,50±0,11	1,60 [1,30;1,60]	1,20	1,80
30th day	1,48±0,16	1,60 [1,10;1,70]	1,10	1,90
90th day	1,56±0,04	1,50 [1,50;1,60]	1,50	1,70
180th day	2,16±0,22	2,00 [1,80;2,40]	1,70	2,90

Supplementary Table S3. Descriptive Statistics for the Control Samples on Each Studied Day and Across All Days Combined.

Indicators	M±SD	Me [25%;75%]	Min	Max
Dermal Thickness, microns	1981,14±89,64	1995,13 [1947,83;2080,66]	1670,60	2211,46
Diameter of Blood Vessels, microns	70,70±11,85	74,35 [58,15;81,62]	34,21	105,15
Relative Vascular Bed Area, %	5,19±1,26	5,23 [2,73;6,50]	2,30	9,17
Fibrocytes, count	4281,00±678,60	4795,00 [4281,00;4966,00]	1712,00	5651,00
Density of Type I Collagen in the Dermis, pixels	71,12±6,22	70,30 [58,30;84,50]	56,60	85,90
Density of Type I Collagen in the Hypodermis, pixels	9,20±0,76	9,10 [8,10;9,80]	7,30	11,70
Density of Type III Collagen in the Dermis, pixels	12,18±2,77	12,90 [6,20;15,00]	6,10	20,70
Density of Type III Collagen in the Hypodermis, pixels	13,70±1,18	13,10 [12,00;15,10]	10,80	17,50
Ratio of Type I/III Collagen in the Dermis	7,95±2,45	5,45 [3,77;13,85]	2,82	13,85
Ratio of Type I/III Collagen in the Hypodermis	0,70±0,10	0,61 [0,56;0,69]	0,54	1,08
Density of Elastin in the Dermis, pixels	1,14±0,10	1,10 [1,00;1,20]	0,90	1,50
Density of Elastin in the Hypodermis, pixels	1,10±0,16	1,00 [0,90;1,10]	0,80	1,70
